# Vital lessons from struggling partnerships and potential partnerships: an international study with leaders across the health sector

**DOI:** 10.1186/s12913-024-11944-7

**Published:** 2024-11-26

**Authors:** Greg Zwisler, Christopher Martin Sauer, David Shoultz

**Affiliations:** 1 Independent researcher, Seattle, USA; 2 Faculty of Medicine, Department of Hematology and Stem Cell Transplantation, University Hospital Essen, University Duisburg-Essen, Essen, Germany; 3 Institute for Artificial Intelligence in Medicine, University Hospital Essen, Giradetstr. 2, Essen, 45131 Germany; 4 Department of Epidemiology, School of Public Health, University of Washington, Seattle, USA

**Keywords:** Interorganizational collaboration, Barriers and facilitators, Intersectoral collaboration, Integrated care, Health policy, Design, Product development, Patient involvement, OECD countries, Population health

## Abstract

**Background:**

Inter-organizational partnerships and collaborations, used here interchangeably, have growing prominence across the health sector. Successful partnerships have received extensive study. However, especially for partnerships including nonprofit partners, limited attention has been given to negative factors that contribute to struggling partnerships, including failed partnerships, and/or impede potential partnerships, including unexplored and undeveloped potential partnerships. This study aimed to explore these across diverse examples of struggling and potential partnerships considered otherwise worthwhile in principle, according to leaders and managers—in 13 countries across Asia-Pacific, EU+, North America—from diverse roles and settings across the health sector. It also aimed to explore success factors they said contributed to successful partnerships.

**Methods:**

Interviews were conducted with 70 practitioners in 13 countries and a wide range of roles and nonprofit, industry, and government settings, including research institutions, across the health sector. Interviews covered their examples of struggling, potential, and successful partnerships; and, factors. Interview data were analyzed inductively, employing thematic network analysis. Comments underlying themes were reviewed regarding the participants concerned to note range (e.g., regions).

**Results:**

Key findings included: (1) the many negative factors and success factors identified as themes; (2) their occurrence across diverse contexts, including different regions and institutional sectors (i.e., nonprofit, industry, government); (3) the complementarity of negative factors and success factors, with each set placing different emphasis on certain topics and negative factors both broadening the overall range of topics and contributing more to literature; (4) the occurrence of most negative factors with both struggling and potential partnerships. The 255 partnerships and potential partnerships discussed included nonprofit (190/255), industry (112/255), and/or government (140/255) partners. Many spanned two different institutional sectors (147/255); 86/255 spanned one; 20/255 spanned three.

**Conclusions:**

The findings suggest three takeaways for practitioners: (1) factors used to consider partnerships should reflect factors from struggling partnerships and/or potential partnerships, plus successful partnerships; (2) negative factors can highlight opportunities to advance partnerships, individually and systematically; (3) practitioners should consider developing frameworks of factors from literature and experience to facilitate judicious consideration of partnerships and inform approaches, lessons drawn, and potential partnerships sought. Struggling and potential partnerships merit scholarly attention.

**Supplementary Information:**

The online version contains supplementary material available at 10.1186/s12913-024-11944-7.

## Background

Many areas of the health sector have witnessed increased use of inter-organizational and related partnerships, observed as important to better health outcomes, diverse, and growing in complexity [1–8]. The ubiquity and variety of partnerships and collaborations, used here interchangeably, underscore their aggregate significance. This includes both the resources and efforts invested and partnerships’ potential contributions to better health outcomes. Myriad partnerships across the health sector organize activity and resources to advance vital goals [1–11]. The World Health Organization provides an example of the ubiquity of partnerships, defining its partnerships as collaborations within and external to it, including alliances, networks, project collaborations, and joint campaigns [8]. Despite their ubiquity, important gaps remain in our knowledge of partnerships.

Successful inter-organizational partnerships and the factors contributing to their success have received extensive study [12–14]. Despite some recognition that partnerships often fail [15] and of challenges partnerships may encounter [2, 4, 16], limited scholarly attention has been devoted to struggling partnerships, including failed partnerships, and contributing negative factors—both in the health sector and more broadly, especially for partnerships involving nonprofit partners alone or alongside others [4, 12, 17, 18]. This corresponds to noted indications of selection bias, survivorship bias, and publication bias across the related literature. A broad review observed prevailing sampling procedures tend to select more or less successful partnerships and omit those that have failed [17]. Reviews of the literature on various genres of health-sector partnerships noted a telling absence of failed partnerships [4] or partnerships with negative outcomes [16] among published studies. Accordingly, even when scholarship has addressed negative factors, it has largely done so by studying relatively successful partnerships. Additionally, a broad meta-review of scholarship on partnerships (government, industry, nonprofit, and cross-sector) highlighted scarce attention has been devoted to understanding the collectivity of negative factors contributing to poor performance, and noted studies’ limitation to narrow contexts [12].

In the present study, ‘struggling partnerships’ denotes partnerships agreed and embarked upon yet characterized by deficiencies/problems, including failed partnerships, partnerships representing close variations on failure, and those otherwise falling well short of their expected potential, in terms reflective of study participants’ descriptions (e.g., terminated, desultory, stymied, half-hearted, fraught, difficult, not working out).

There is an additional gap in the literature. Limited attention has been given to what negative factors impede potential partnerships. In the present study, ‘potential partnerships’ denotes all those, notional or otherwise not yet agreed and embarked upon, including: (1) those left unexplored in the first place, and therefore scarcely or not discussed with possible partners, termed ‘unexplored’; (2) those that had their exploration or development terminated, termed ‘abandoned’; (3) those otherwise undeveloped, termed ‘undeveloped’; and, (4) those under exploration or otherwise in development, termed ‘under exploration or development’.

Topics such as unexplored and undeveloped potential partnerships can seem vague: however, they can contribute directly to understanding what partnerships should exist but do not, and why, for example. Yet the literature has scarcely addressed unexplored and undeveloped potential partnerships and what negative factors impede their exploration and development in cases considered otherwise worthwhile by practitioners. Few studies have approached these topics, and then only partly. Illustratively, one such study [19] focused on a program-specific setting and perceptions of risks in the sorts of potential partnerships that practitioners expected based on the program and previous programs. Meanwhile, scholars have tended to overlook the potential partnerships that practitioners might want instead, for example.

Additionally, potential partnerships under exploration or development have received limited and uneven attention. A review of literature on a key genre of health partnerships called for research on potential partnerships and formation, as almost all studies concerned only implementation of partnerships [4].

More broadly, boundary-spanning partnership research conducted across different contexts has been called for to balance the concentration of partnership scholarship within siloes focused on particular contexts, forms, cross-sectoral areas, countries, and academic fields [17, 18, 20].

Practitioners would be better served if the multiple literature gaps described above received more scholarly attention. These interrelated deficits inspired the present exploratory study and its aims.

### Aims

This study primarily aimed to explore negative factors that contribute to struggling partnerships and/or impede potential partnerships, according to practitioners across the health sector. Specifically, it aimed to identify negative factors thematically in relation to a broad range of examples of struggling partnerships and potential partnerships raised individually by leaders and managers (i.e., practitioners) drawn from 13 mainly OECD countries across Asia-Pacific, EU+, North America—and diverse roles in a wide array of nonprofit, government, and industry settings across the health sector. It concerned struggling partnerships, including failed partnerships, and potential partnerships, including unexplored and undeveloped potential partnerships, that participants considered otherwise worthwhile in principle. The preceding section defines struggling partnerships, potential partnerships, and related terms. This study also aimed to explore success factors contributing to successful partnerships, according to the same practitioners. Specifically, it aimed to identify success factors thematically in relation to a broad range of examples of successful partnerships they raised. Additionally, it aimed to broadly compare the success factors and negative factors identified. This study also aimed to note the range of regions and institutional sectors each of the factors surfaced across, regarding participants whose comments contributed to the corresponding themes, and for negative factors, whether those comments concerned struggling or potential partnerships, or both. To identify negative factors and success factors thematically as just outlined, this study aimed to use thematic network analysis of participant interview data, with an inductive approach.

### Theoretical context: partnerships and collaborations

This section helps relate the present study to fields of scholarship. This study used the terms partnerships and collaborations interchangeably for ease of understanding by practitioners as participants and a vital audience. It addressed partnerships that were collaborative activities synonymous with collaborations and excluded other situations such as mergers. It focused on inter-organizational partnerships, defining these consistent with both the foregoing and what scholars refer to as inter-organizational collaborations (IOCs). Theoretical definitions of IOCs abound. Here IOC is used with reference to the broad theoretical delineations summarized by [4], particularly, and other conceptions such as [21] to the extent these are compatible.

## Methods

This exploratory qualitative study employed semi-structured interviews. Additional file 1 provides further methodological detail, including both the interview guide and the version of the interview guide shared with participants that were developed for this study (Additional file 1, pages 8–10).

### Participants and interviews

Through purposeful sampling [22] oriented to the health sector broadly, interviews were conducted with 70 participants—in 13 countries across Asia-Pacific, EU+, and North America—who were leaders and managers in a wide range of different roles and nonprofit, industry, and government settings, including research institutions (Table 1). Countries were OECD members, except for one OECD key partner. Participants were recruited from a wide array of roles and areas across the health sector (e.g., clinical quality, health system leadership, pharmaceutical R&D, population health, advocacy, digital transformation) and different levels of seniority (Table 1). All participants were based within the health sector except two discussed below. Table 1 provides a detailed summary of the participants and an anonymized list of their disguised titles and organizations grouped by institutional sector.

**Table 1:** Study participants

**General**	**All participants**: *n*=70; female (*n*=32), male (*n*=38).
	Seniority by roles*:chairs, CEOs, top executives (*n*=20, thereof 10 for organizations <250 staff); other C-level executives, vice presidents, directors, similar (*n*=36); associate directors, managers, similar (*n*=11); professors, others (*n*=7).
Institutional sectors and organizational settings overview*	**Government **(*n*=22): health ministries and counterpart bodies (*n*=7); regional health systems (*n*=5); hospitals (*n*=6); innovation support agencies (*n*=3); applied research, disease control (*n*=1).
	**Industry** (*n*=23): pharmaceuticals (*n*=9); medtech (*n*=3); digital health startups (*n*=2) and investing (*n*=2); insurance (*n*=3); community-based services (*n*=3); hospitals (*n*=1).
	**Nonprofit** (*n*=29): associations of healthcare organizations (*n*=2) and professionals (*n*=4); hospitals (*n*=4); community clinics (*n*=2); academic institutions (*n*=3); insurance (*n*=1); nonprofits focused on: global health (*n*=4), patient-focused care (*n*=3), specific diseases (*n*=2), or facilitation of other innovation and change (*n*=4).
	**Size of organization**: very large, 10,000+ staff (*n*=21 participants, median 50,000 staff); large, 250-9,999 staff (*n*=28, median 2,475 staff); small and medium, <250 staff (*n*=25, median 20 staff).
**Areas of focus and leadership overview**^†^	**Health services delivery systems, operations, innovation and improvement** (*n*=39): public healthcare system, national apex (*n*=4), regional apex (*n*=3); hospital, apex (*n*=7); community-based services (*n*=12); population health and social determinants of health, program development and applied research (*n*=8); digital transformation of healthcare (*n*=8); service innovation support units in healthcare settings (*n*=6); clinical quality and improvement (*n*=6).
	**Pharmaceutical, digital health and medtech product development, related activities and access **(*n*=30): pharmaceutical R&D (*n*=6); digital health and medtech R&D (*n*=3); product planning, business development and adoption support (*n*=6); market/non-market financing and facilitation for development of products, related startups (*n*=9); institutional management of R&D projects with industry (*n*=3); domestic and global product access (*n*=7).
	**Health systems policy and implementation, finances, and private insurance **(*n*=23): government health system policy development, implementation and finances in executive (*n*=4) and technical (*n*=3) areas of health ministries and counterpart bodies; external applied research, expert policy advice (*n*=4); policy advocacy, for healthcare organizations, professionals, patients, et al (*n*=8); health-financing transformation (*n*=3); private health insurance and insurers’ new initiatives (*n*=5).
	**Patient voice and involvement** (*n*=6): advocacy and representation (*n*=3); patient-focused care transformation capacity (*n*=3).
	**Health professionals** (*n*=9): associations of health professionals (*n*=4); active clinical practice in addition to other roles (*n*=6).
	**Marginalized populations and areas** (*n*=21): lower-socioeconomic, domestic (*n*=6); global health (*n*=10); mental health (*n*=6).
**Locations overview, by region**	**Asia-Pacific** (*n*=21) : Australia (*n*=8); Japan (*n*=4); Singapore (*n*=7); also, beyond the main focus on OECD countries, select interviews were done in China (*n*=2).
	**EU+** (*n*=34): France (*n*=3); Germany (*n*=3); Netherlands (*n*=3); Sweden (*n*=6); Switzerland (*n*=7); United Kingdom (*n*=6); and, Israel (*n*=6; grouped with EU+ region to facilitate anonymity).
	**North America** (*n*=15): Canada (*n*=3); United States (*n*=12).
**Government participants**	Anonymized and disguised titles/organizations for each participant: · Chief of staff, executive office responsible for public healthcare delivery, ministry of health · First assistant secretary, community-based care, ministry of health · Chief medical information officer, digital transformation, ministry of health · Director, secretariat to policy and service coordination body for national healthcare system · Assistant deputy minister, healthcare portfolios, regional ministry of health · Associate national medical director, chronic disease, national healthcare system · CEO, regional healthcare system · Associate director, health and life sciences division, national innovation agency · Policy development team lead, health policy area, inter-governmental health agency · Director, hub and network for clinical service design, regional healthcare system · Director, clinical quality and safety, specialized major teaching hospital · Director for portfolio and partnerships management, innovation hub, major teaching hospital · Chief community integration officer, regional healthcare system · CEO, regional health innovation network · Program officer, health systems and services, inter-governmental health agency · Associate director, innovation hub, major teaching hospital · Director, health technology innovation accelerator and small-medium enterprise fund · Head, medical device innovation program, major teaching hospital · Head, design and innovation, quality and care improvement, regional healthcare system · Director, major teaching hospital · Director, national disease control research institution · Chief clinical officer and chief innovation officer, major teaching hospital
**Nonprofit participants**	Anonymized and disguised titles/organizations for each participant: · CEO, major association of health professionals · CEO, national association for patient representation and service reforms across health system · Chair, health product development and access nonprofit, and applied health research leader · President, national disease society · Director of strategic initiatives and clinical advisor, major association of health professionals · Country head, medtech incubation and novel health finance, think tank with catalyst programs · Executive director, national association of healthcare organizations · Executive director, community clinic system · President, special interest group, major association of health professionals · Head, strategy and innovation, regional nonprofit with focus on healthcare transformation · CEO, community hospital, regional healthcare system · Board director, regional health insurer · Director of outreach and population health services, group of community clinics · Head of industry partnerships, major teaching hospital · Professor, national advocate of co-designed digital/clinical healthcare for neglected populations · Policy director, national health services group, major association of healthcare organizations · Executive director, nonprofit with focus on patient-centered care · CEO, regional nonprofit with focus on patient representation across healthcare system · Lead, network initiatives and human capital for change, innovation hub, major teaching hospital · Strategy director, catalyst program to shift health spending toward equitable population health · Director, clinical quality improvement collaborative, disease foundation · Head, health facilities and adaptive housing, design standards development nonprofit · Chief investment officer, social and health catalyst fund · Director, specialized teaching hospital · General manager, national association of health professionals · CEO, health product development catalyst fund · Professor and head, healthcare governance research unit, major university · Associate director, area initiatives and supply chains, health and development catalyst fund · Chair, specialized community hospital
**Industry participants**	Anonymized and disguised titles/organizations for each participant: · Vice president, global lead, specialty area clinical development, major pharma multinational · CEO, business unit, major health insurer · CEO, connected health services business, and digital strategy lead, national home care company · Global lead, several diseases, early research and development, major pharma multinational · Director, national program development, population health, major health insurer · Vice president, global health initiatives, major pharma multinational · Senior manager, strategy and healthcare transformation, major health insurer · Chief medical officer, digital therapeutics startup · Director, program development and innovation, national occupational health services company · Director, public healthcare systems, global policy and access, major pharma multinational · Vice president, product, digital health startup · CEO and founder, medical device startup · Board director, nationwide assisted living and dementia care company · Country lead, research and open innovation, major pharma multinational · Board member and investor, digital health startups, and startup community leader · Vice president, health data science area, major pharma multinational · Principal manager, product development portfolio management, major pharma multinational · Principal, portfolio development and partnerships, corporate venture capital, health insurer · Vice president, national medical affairs, major pharma multinational · Regional director, technology marketing, major medtech multinational · Consulting manager, implementation and services, major medtech multinational · Senior director, global health, major pharma multinational · CEO, community hospital

*Indicates subtotals under these headings add to 74 since three participants held and spoke to multiple roles and affiliations

^†^Indicates subtotals within this section do not add since many participants were involved in more than one of the 27 areas listed (median=2; range=1-5)

Participants were recruited on a rolling basis to support representation of diverse settings, roles, and regions, congruent with study aims. Recruitment, by email/LinkedIn messages to individually-selected invitees, drew especially from outside of author networks (*n* = 52) with identification by internet searches (40/52) and selective use of snowballing referrals (12/52). Identification by internet searches drew on sources such as local health-sector institutions’ leadership directories and their press releases on various topics that mentioned key personnel involved, searches of local LinkedIn members in health-sector fields, and websites of health-sector associations. Recruitment also made selective use of authors’ networks (*n* = 8) and referrals from authors’ networks (*n* = 10). All participants were based within the health sector except two in a closely related field, recruited from authors’ networks, whose work had long included health initiatives with health groups. Individuals were excluded from the study if in sales or standard support areas such as human resources. Also, individuals were excluded if they indicated they could not speak to partnership topics in the interview guide sent in advance of interviews; in such cases, individuals emailed their regrets. Recruitment and interviews were conducted in English.

Interviews of 28–80 min (mean = 52min; median = 53min) covered examples of struggling partnerships and potential partnerships corresponding to the aims above, negative factors in each, examples of successful partnerships, and success factors in the latter. Participants volunteered all examples. The interview guide defined partnerships broadly as informal or formal collaborations and provided criteria applicable to all examples, including: (a) not simply a transaction or vendor-client relationship; (b) relevant to participants’ current role or, for examples from their past, to their role then; (c) relatively distinctive from the participant’s perspective; and, (d) health-related, i.e. supportive of better health outcomes and ideally access. The first example discussed was a successful partnership: a partnership experience the participant considered a successful partnership and productive, and felt enthusiastic about. Interviews then inquired into examples of struggling partnerships and potential partnerships. Interviews were conducted by GZ from February to July 2017 (*n* = 53) and August to November 2018 (*n* = 17), with the interim pause due to other responsibilities (manuscript preparation was later delayed due to the coronavirus pandemic). Interviews were recorded and transcribed, except for 4 participants who preferred only notes be taken.

### Analysis

After data collection was complete, interview data were analyzed thematically employing principles of thematic network analysis [23], following the approach used in [24] as further discussed in Additional file 1. This approach was attractive thanks to its flexible support for: development of an organized set of themes, or thematic network, around a global theme matching a research/interview question; a degree of interpretation and data reduction appropriate to this study’s aims and themes’ accessibility to practitioners; use of tables to illustrate each set of themes; and, the iterative analytic process. Accordingly, separate thematic networks were developed around (1) negative factors and (2) success factors. For each of these two global themes, a set of themes grouped under organizing themes was iteratively derived, refined, and applied across all transcripts. Theme identification used inductive coding. Analysis was supported by Nvivo software (Lumivero, Denver) and did not use auto-coding. The comments underlying each of the finalized themes were reviewed regarding the participants concerned to note range, specifically, study regions and institutional sectors represented, as well as whether participants were concentrated in one area or field. For negative factors, underlying comments were also reviewed as to whether they had been made only in discussions of struggling partnerships or of potential partnerships.

## Results

This section presents key findings, foremost the negative factors identified, then success factors, and also broadly compares both sets of factors. Negative factors often related to broader circumstances, and collectively extended beyond the range of topics touched on by success factors, which concentrated more on partnerships’ management and key individuals. To help contextualize the study findings this section also summarizes the overall breadth of partnership examples that participants shared and discussed.

### Negative factors in struggling partnerships and potential partnerships

Analysis of negative factors raised by participants identified 36 themes grouped under ten organizing themes (see Table 2 for the complete list of themes). Notably, themes generally each surfaced across different regions and institutional sectors (Table 2). Moreover, they generally also surfaced in connection both to struggling partnerships and potential partnerships. Organizing themes are presented below with summaries reflective of the themes grouped under them. Additional file 2 provides illustrative quotes for each theme, featuring 143 illustrative quotes from 62 participants.

**Table 2:** Negative factors

Organizing themes	Themes	Regions^a^	Sectors^b^
Problematic health-sector structures and mentalities	Health finance structures, the ways money flows, their flaws and effects	E,A,N	N,G,I
	Prevailing mental models and general inertia of: health systems, health policy, medical culture, routine clinical delivery	E,A,N	N,G,I
	Extensive rules and regulations	E,A,N	N,G,I
	Paternalism, traditional mindsets limit meaningful patient involvement	E,A,N	N,G
	Insularity, not going outside health sector or home country to learn from, work with others	E,A,N	N,G,I
Misaligned motivations and competitive dynamics	Misaligned underlying interests, aims, incentives of those involved	E,A,N	N,G,I
	Competition, partners’ needs to prioritize their own competitiveness	E,A,N	N,G,I
Deficiencies in leadership, partnership management, and communication	Lack of strong leadership	E,A,N	N,G,I
	Lack of partnership skills, experience	E,A,N	N,G,I
	Deficiencies in communication practices, mechanisms, skills	E,A,N	N,G,I
	Objectives, activities insufficiently focused, not clearly defined	E,N	N,G,I
	Not adequately understanding partners, their needs	E,A,N	N,G,I
	Alignment not given more probing, careful attention upfront	E,N	N,I
Alignment time-consuming to establish and uncertain	Negotiations, alignment building, contracting involved seen as: difficult, prolonged, vulnerable to failure, too time and resource intensive	E,A,N	N,G,I
	Cumbersome pace, processes of universities, major corporations, government bodies	E,A,N	N,G,I
	Turnover of senior leaders, partnership leads, liaisons at partners	E,A,N	N,I
	Too many partners involved	E,N	N,G,I
	Resistance, limited cooperation from within partner's organization and structure	E,A,N	N,G,I
	Difficult for government bodies to collaborate with each other	E,N	G,I
Challenges of efforts involving newer or less familiar areas and approaches	Partners lack competencies, capabilities vital to their roles, the partnership	E,A,N	N,G,I
	Risk-averse attitudes, people afraid to try something new	E,A,N	N,G,I
	Business models uncertain, yet to be determined, inadequate	E,A,N	N,G,I
	Interested and suitable partners hard to identify, not aware of each other	E,A,N	N,G,I
	To yield results only in longer term with notable uncertainties, upfront investments involved	E,A,N	N,G,I
Limited overall resources and influence of partners, funding hard to come by	Limited resources of partners’ own organizations	E,A,N	N,G,I
	Hard to come by funding for the partnership	E,A,N	N,G,I
	Partners not influential enough, too small, relatively unimportant	E,A,N	N,G
Value unrealized or unclear to some concerned	Value unrealized, intangible, not apparent to some concerned	E,A,N	N,G,I
	General sphere of activity not a priority to some concerned, including partnerships related to it	E,A,N	N,G,I
Relationships negative or insufficiently developed	Relationships insufficiently developed, limited in part by the time involved to cultivate them and related practicalities	E,A,N	N,G,I
	Mistrust, insufficient trust	E,A,N	N,G,I
	People involved do not get along, work well together	E,A	N,G,I
	Treated like a vendor rather than a partner	E,A,N	N
Lack of time given other work demands	Lack of time given other work demands	E,A,N	N,G,I
Industry and government partners face hurdles to joint collaboration	Hard for industry and government partners to engage, work together	E,A,N	N,G,I
	Skepticism of industry partners	E,A,N	G,I

^a^Key to Regions: E=EU+; *A *Asia-Pacific, *N *North America

^b^Key to Sectors: *N *Nonprofit, *G *Government, *I *Industry

The set of negative factors identified was diverse and wide-ranging (Table 2). Negative factors often related to broader circumstances, namely: those within organizations, those tied to basic aspects of the partnerships or kinds of partnerships discussed, and/or those in the institutional environment.

#### Problematic health-sector structures and mentalities

Participants pointed to persistent limiting attitudes, concepts, policies, and practices within the health sector, and their effects, in terms of: health finance structures and their influence; prevailing mental models and inertia in areas from health systems and policy to medical culture and routine clinical delivery; extensive rules and regulations; paternalism and traditional mindsets limiting meaningful patient involvement; and, insularity.

#### Misaligned motivations and competitive dynamics

Participants pointed to differences and conflicts in the underlying interests, aims, and incentives of those involved, including whether they were effectively competitors, and described these as misaligned features of the context and parties. They explained how these differences limited particular partnerships and cooperation generally.

#### Deficiencies in leadership, partnership management, and communication

Participants cited inadequate skills, abilities, and practices around leadership and management relative to partnerships and the work they aimed to advance, including with respect to: communication, objectives, understanding partners or potential partners, and attention to matters of alignment. Some aspects of deficiencies in leadership, partnership skills, and communication extended to the institutional environments concerned.

#### Alignment time-consuming to establish and uncertain

Participants cited the difficulty and amounts of time and negotiation involved to try to achieve alignment and agreements, how alignment could nevertheless prove elusive or ephemeral, and contributing features such as: cumbersome pace and processes of various partners; turnover of senior leaders or partnership leads and liaisons at partners; too many partners; limited cooperation from within partners; and, the difficulty of forging collaboration among government bodies, as well as different interests (the latter were reflected by ‘Misaligned motivations and competitive dynamics’as to areas of underlying misalignment).

#### Challenges of efforts involving newer or less familiar areas and approaches

Participants pointed to problems such as: risk-averse attitudes to trying something new, partners’ lack of needed competencies and capabilities, difficulties identifying interested and suitable partners, uncertain business models, and upfront investments for only longer-term and uncertain results. These problems were noted mainly for partnerships aimed at newer areas or ones less familiar to those involved.

#### Limited overall resources and influence of partners, funding hard to come by

Participants pointed to partners’ own constrained organizational resources, and limited relative influence and importance. They also noted challenges raising grant funding when partnerships required this.

#### Value unrealized or unclear to some concerned

Participants raised limitations to do with partnerships’ value or potential value, such as when value was unrealized, intangible, or not apparent to some parties involved. Participants also cited examples when the general sphere of activity, including partnerships related to it, was not a priority for some parties or within their usual scope.

#### Relationships negative or insufficiently developed

Participants cited limitations to relationships and trust. These had either a neutral sensibility, as when there simply had not been opportunity to get to know each other well enough, or a negative sensibility, such as mistrust, personal conflicts, and being treated as less than a partner.

#### Lack of time given other work demands

Participants pointed to how they or counterparts were too busy with their immediate responsibilities and work to develop or manage partnerships noted as otherwise desirable or even necessary.

#### Industry and government partners face hurdles to joint collaboration

Participants cited problems faced by partners from government and industry in terms of difficulties working together, both generally and due to issues, such as lack of common understanding and forums, or tensions around public procurement. They also cited skepticism of industry partners, mainly in government institutions.

As mentioned above, themes generally each surfaced across different regions and institutional sectors. The comments underlying each theme spanned comments by participants from all three institutional sectors (29/36 themes) or two of three sectors (6/36 themes), except only nonprofit participants raised one theme (Table 2). Similarly, they spanned comments by participants in all three regions (31/36 themes) or two of three regions (5/36 themes) (Table 2). In no themes were the underlying comments found to have all come from participants who worked in the same area as each other (e.g., hospitals, pharmaceuticals, global health).

The comments underlying each theme spanned comments on examples of struggling partnerships and comments on examples of potential partnerships (33/36 themes) with the following three exceptions. Only comments on examples of potential partnerships contributed to ‘Interested and suitable partners hard to identify, not aware of each other’. Meanwhile, only comments on examples of struggling partnerships contributed to either ‘Treated like a vendor rather than a partner’ or ‘Alignment not given more probing, careful attention upfront’.

### Success factors in successful partnerships

Analysis of success factors raised by participants identified 24 themes grouped under seven organizing themes (see Table 3 for the complete list of themes). Notably, themes generally each surfaced across different regions and institutional sectors (Table 3). Organizing themes are presented below with summaries reflective of the themes grouped under them. Additional file 3 provides illustrative quotes for each theme, featuring 89 illustrative quotes from 47 participants.

**Table 3:** Success factors

Organizing themes	Themes	Regions^a^	Sectors^b^
Meaningful value for and from partners, responsive to key needs	Win-win, partners respectively see benefits they value	E,A,N	N,G,I
	Partners respectively bring value, complement each other	E,A,N	N,G,I
	Identify and respond to the right needs, proactively	E,A,N	N,G,I
	Addresses pressing needs and challenges motivating partners, felt by them	E,A,N	N,G,I
	Collaboration facilitated by partner’s well-adapted size, structure	E	N,G,I
Shared purpose and commitment, around humanitarian aims	Shared purpose, resolve	E,A,N	N,G,I
	Personal involvement in humanitarian shared purpose	E,A,N	N,G,I
Professional relationships and trust, their cultivation and good basis	Professional relationships, history with each other and accrued trust	E,A,N	N,G,I
	Actively build trust, relationships	E,A,N	N,G,I
	Collaboration as peers and equals, of professional peers	E,A,N	N,G,I
Strategic vision, support, and approach	Strategic vision and approach	E,A,N	N,G,I
	Senior leaders provide their support, clear mandate	E,A,N	N,G,I
	Key financial commitments signal credibility, provide resources	E,A,N	N,G,I
	Effectively develop vital supporting partnerships, support of key stakeholders, internally and externally	E,A,N	N,G,I
The right people, individuals make the difference	The right people, particular individuals’ involvement as pivotal	E,A,N	N,G,I
	Leadership by key individuals	E,A,N	N,G,I
	Personal affinity, working together well	E,A,N	N,G,I
	Key individuals’ experience in professional spheres the partnerships inhabit	E,A	N,G,I
Open communication, partners upfront and understand other partners’ perspectives	Partners upfront about their respective objectives, expectations, concerns	E,A,N	N,G,I
	Active communication, information exchange	E,A,N	N,G,I
	Understanding where other partners are coming from	E,A,N	N,G,I
	Useful convenings of partners, regular check-ins	E,A,N	N,G,I
Active management, from early on	Assiduous management efforts to set the stage for success, to follow through and deliver	E,A,N	N,G,I
	Clearly define how partners will work together, joint objectives	E,A,N	N,G,I

^a^Key to Regions: *E*=EU+, *A *Asia-Pacific, *N *North America

^b^Key to Sectors: *N *Nonprofit, *G *Government, *I *Industry

The set of success factors identified represented a variety of considerations (Table 3). Overall, it was narrower than the set of negative factors: a broad comparison follows further below. Success factors often related to aspects of management, leadership, and/or people involved.

#### Meaningful value for and from partners, responsive to key needs

Participants cited mutual benefit, how partners respectively see benefits they value and bring value, such as key complementary capabilities, and how a partner’s well-adapted size and structure can make them easier to work with productively. They also pointed to when the focus of effort addresses partners’ challenges or, relatedly, reflects astute identification of needs.

#### Shared purpose and commitment, around humanitarian aims

Participants pointed to partners’ common ambition and dedication regarding (larger) aims advanced by their partnership, and to how this aided and even characterized the partnerships in these cases. They pointed to when the shared purpose was humanitarian, such as improving access or patient outcomes, and people felt personally committed to it.

#### Professional relationships and trust, their cultivation and good basis

Participants pointed to familiarity and positive history of people with each other and well-developed relationships and trust. They also pointed to efforts to build trust and relationships from early on if people were newer to each other, and to collaboration with professional peers or in the spirit of being peers and equals.

#### Strategic vision, support, and approach

Participants cited a strategic vision and approach, judicious and effective, in terms of compelling overall plans and guiding aims pursued systematically. Relatedly, they pointed to skillful development of stakeholder buy-in and supporting partnerships internally and externally. Participants also cited strategic support, mandates and credibility provided by backing from senior leaders and key financial commitments.

#### The right people, individuals make the difference

Participants described particular individuals’ involvement as pivotal, noting how if others had been involved instead things could have gone poorly. Relatedly, they also cited leadership by key individuals, personal affinity, and key individuals’ experience in professional spheres inhabited by the partnerships.

#### Open communication, partners upfront and understand other partners’ perspectives

Participants cited straightforward sharing by partners of their own objectives and expectations or other potentially sensitive concerns, active communication, regular check-ins, and useful in-person convenings. They also cited understanding where other partners are coming from, through thoughtful listening and insight.

#### Active management, from early on

Participants pointed to assiduous management efforts to set the stage for success and follow through, and to clear definition of objectives and how partners will work together.

The comments underlying each theme spanned comments by participants in all three institutional sectors and, for 22/24 themes, all three regions (Table 3). In no themes were the underlying comments found to have all come from participants who worked in the same area as each other (e.g., hospitals, pharmaceuticals, global health).

### Broad comparison of negative factors to success factors

Broad comparison of negative factors to success factors indicates that while the two sets of factors partially overlapped in terms of topics they touched on, the range and nature of topics also diverged. Negative factors often related to broader circumstances and collectively went well beyond the range of topics that success factors touched on. Notably, about half of negative factors addressed topics not touched on by success factors. The other negative factors together touched broadly on most topics success factors collectively addressed. Success factors often related to aspects of management, leadership, and/or people involved. The following expands on the main points above.

About half of negative factors related to topics not addressed by success factors. These included all or most themes under: ‘Problematic health-sector structures and mentalities’; ‘Alignment time-consuming to establish and uncertain’; ‘Challenges of efforts involving newer or less familiar areas and approaches’; and, ‘Industry and government partners face hurdles to joint collaboration’.

The other negative factors together touched broadly on most topics success factors collectively touched on. Both sets of factors thus touched on several broad topics including: value, what the different partners bring, alignment, aims, trust, relationships, leadership, support, management, people, communication, and understanding. Overall, success factors often related to aspects of management, leadership, and/or the people involved (e.g., ‘The right people…’, ‘Strategic vision and approach’, ‘Identify and respond to the right needs, proactively’, ‘Actively build trust, relationships’).

Negative factors often related to broader circumstances, namely: those within organizations, those tied to basic aspects of the partnerships or kinds of partnerships in question, and/or those in the institutional environment (e.g., ‘Difficult for government bodies to collaborate…’, ‘Challenges of efforts in newer or less familiar areas…’, ‘Limited resources of partners’ own organizations’, ‘Lack of partnership skills, experience’, ‘Extensive rules and regulations’, ‘Skepticism of industry partners’). Many of these related to topics not addressed by success factors.

### Context: breadth of participants’ partnership examples

This section summarizes the breadth of partnership examples given by participants, thus providing broad context for the negative factors and success factors presented above. In the following, the counts of examples do not represent the prevalence of partnerships; this study did not investigate prevalence.

A total of 255 examples of partnerships (*n* = 131) and potential partnerships (*n* = 124) were mentioned. These were mainly inter-organizational (*n* = 249), comprised of dyadic examples with 2 partners (*n* = 148) and non-dyadic examples (*n* = 101) with 3–4 partners (*n* = 39) or ≥ 5 partners (*n* = 62); the remainder were intra-organizational within large and very large organizations (*n* = 6). Examples with ≥ 1 partners from each of two institutional sectors were common (*n* = 147) and featured: government and nonprofit partners (*n* = 72); industry and nonprofit partners (*n* = 53); and, government and industry partners (*n* = 22). Single-sector examples were also common (*n* = 86: nonprofit = 43; government = 26; industry = 17). Several examples included all 3 sectors (*n* = 20). Overall, government partners featured in 140/255 examples, nonprofit partners in 190/255 examples, and industry partners in 112/255 examples: roles varied. In two examples of a particular nonprofit’s partnerships, both dyadic, the other partners’ profiles and sectors were withheld.

The wide variety of partnership examples discussed broadly corresponded to the participants concerned (e.g., pharmaceutical R&D executives discussed partnerships related to pharmaceutical R&D). Participants’ areas of focus, settings, and roles have been described anonymously in some detail (Table 1) partly so readers may gain an overall sense for the areas partnerships involved.

Overall, examples of struggling partnerships and/or potential partnerships were given by 69/70 participants, of whom 68 mentioned negative factors which contributed to ≥ 1 of the themes identified.

Examples of potential partnerships were given by 67 participants and comprised: unexplored and undeveloped potential partnerships they wished would transpire (46 participants); abandoned potential partnerships (9 participants); and, potential partnerships under exploration or development (34 participants). With unexplored and undeveloped potential partnerships the prevailing sense was of potential value but considerable negative factors, alongside limited current impetus and/or possibility to overcome those factors; some were described more sanguinely. Among potential partnerships under exploration or development, expectations varied and negative factors appeared either considerable or somewhat considerable in most.

Examples of struggling partnerships, given by 48 participants, included failed partnerships, partnerships representing close variations on failure, and those otherwise falling well short of their expected potential (e.g., terminated, desultory, stymied, half-hearted, fraught, difficult, not working out). Most had been terminated, run their course in an attenuated state, or nominally continued, and were noted for their unrealized potential. Participants who did not give examples of struggling partnerships instead responded with germane examples of potential partnerships (15 participants), including abandoned potential partnerships, or were not asked due to time (4 participants).

Examples of successful partnerships were given by 60 participants, all of whom credited success factors which contributed to ≥ 1 of the themes identified.

## Discussion

This section highlights the present study’s uniqueness, its most important findings, and suggested takeaways for practitioners. It then discusses those takeaways before covering additional topics and contributions.

This study explored negative factors that contribute to struggling partnerships and/or impede potential partnerships in the health sector. It focused on inter-organizational partnerships including and not limited to partnerships involving nonprofit partners, as well as industry and/or government partners. Additionally, it explored success factors participants credited for contributing to successful partnerships. This study is the first to explore negative factors in partnerships by examining a wide range of struggling partnerships, including failed partnerships. It is also the first to do so by examining a wide range of potential partnerships, including those left unexplored and undeveloped. More broadly, this is the first study to explore negative factors and success factors thematically across a broad range of health-sector settings, partnerships, and practitioners—as well as several different countries. This exploratory study and its pioneering scope responded to gaps in the literature described above.

This novel study’s most important findings were: (1) the many negative factors and success factors identified as themes; (2) their occurrence across diverse contexts, including different international regions and institutional sectors (i.e., nonprofit, industry, government); (3) the complementarity of negative factors and success factors, with each set placing different emphasis on certain topics and negative factors broadening the overall range of topics; and, (4) the occurrence of most negative factors with both struggling partnerships and potential partnerships.

The findings suggest three takeaways for practitioners, discussed further below, namely: (1) factors used to consider partnerships should reflect factors from struggling partnerships and/or potential partnerships, plus successful partnerships; (2) negative factors can highlight opportunities to help advance partnerships, individually and more systematically; and, (3) practitioners should consider developing frameworks of factors from literature and experience to facilitate judicious consideration of partnerships and inform approaches, lessons drawn, and potential partnerships sought.

Success factors and negative factors were complementary overall. This suggests organizations should draw on factors derived from struggling partnerships and/or potential partnerships, as well as factors derived from successful partnerships, and apply these in combination to their consideration of partnerships. Collectively, negative factors covered a far greater range of topics than success factors and at least partly overlapped most topics covered by the latter, with differences in emphasis. About half of negative factors concerned topics not addressed by success factors. For their part, almost all success factors at least partly overlapped with some negative factors, regarding topics themes touched on. Overall, however, the two sets of themes—negative factors and success factors—also clearly emphasized some different topic areas. More success factors highlighted key individuals involved and/or aspects of partnerships’ leadership and management. Meanwhile, more negative factors concerned broader circumstances: within organizations; tied to basic aspects of the partnerships or kinds of partnerships in question; or, in the institutional environment. The differences between negative and success factors suggest considering both in combination for a more multi-faceted and complete perspective in practice.

Negative factors such as those identified by this study serve as cautions yet can also highlight opportunities to help advance partnerships, individually and more systematically. These opportunities come across three broad levels, illustrated as follows.

First, at the level of a particular partnership, recognizing factors relevant to the partnership and adjusting accordingly may be helpful for any of the partners. For example, when the partnership involves newer areas, the potential negative factors headlined by ‘Challenges of efforts involving newer or less familiar areas and approaches’ may inspire corresponding adjustments at partners and/or jointly among them. These might proactively address implications for expectations and planning, allocation of skilled liaisons, and fair performance evaluation of personnel and the partnership, for instance. Similarly, ‘Risk-averse attitudes, people afraid to try something new’ may inspire mitigations such as starting small.

Second, an organization may find considering negative factors helps highlight opportunities to strengthen its partnership efforts generally. For example, if it recognizes potential negative factors under ‘Alignment time-consuming to establish and uncertain’ these may inspire it to mitigate them by modifying its structures and practices. For instance, risks to alignment from changes to senior leaders such as CEOs, flagged as a negative factor in this study by ‘Turnover of senior leaders…’, could inspire an organization to implement practices that make its commitments to its partnerships more resilient to its leadership changes. Over time, this may enhance its reputation and value as a partner, and may thereby also reduce risks from leadership changes at its partners. Alternatively, other negative factors, such as ‘Partners not influential enough, too small…’, might help inspire smaller organizations to consolidate.

Third, funders and other organizations encouraging partnerships may find negative factors highlight opportunities to modify aspects of the institutional environment to make it more fertile ground for partnerships. For instance, the negative factor this study identified around insularity in the health sector might inspire, for example, support for clinical-safety innovation partnerships to involve international collaboration and safety-centered professionals from outside the health sector. Similarly, negative factors suggesting partnership efforts can face particular challenges in the government sphere, such as ‘Difficult for government bodies to collaborate…’, could help inspire efforts to enhance partnership capabilities within government.

Frameworks used in organizations to help assess partnerships and/or potential partnerships carry practical significance, including when those in use are ad hoc or even unwritten. They can directly affect approaches taken with partnerships, lessons drawn, and potential partnerships sought. This study suggests practitioners, to facilitate judicious consideration of partnerships, should consider developing their own frameworks of factors from literature and experience. Frameworks can serve as aide-mémoires, listing potential factors, to help avoid overlooking relevant factors in a case: this seems advisable given the abundance of potential factors. This study reinforces and expands on previous reports of myriad potential factors [2, 4, 6, 16, 25–28]. It suggests many negative factors affect different struggling partnerships, and potential partnerships, in diverse contexts—possibilities previously unexplored [4, 12, 17]. Practitioners developing frameworks can include potential factors inspired by research and experience they consider pertinent in their own organization’s unique circumstances, for instance. Auschra [4] emphasizes the inter-relatedness of negative factors, advises assuming visible factors may be driven or influenced by factors less immediately obvious, and suggests looking for such related factors in any case. Practitioners, when identifying potential factors from their own experience and that of their organizations, should consider delving into experiences with struggling and potential partnerships. This appears advisable given literature limitations, the complementarity of negative and success factors in this study, and that many negative factors it identified were previously unrecognized.

About one third of negative factors were previously not recognized in the literature, generally or with respect to some of their main aspects. These included most themes under ‘Alignment time-consuming to establish and uncertain’ and ‘Challenges of efforts involving newer or less familiar areas and approaches’, plus others, including those on paternalism and insularity under ‘Problematic health-sector structures and mentalities’ (Additional file 4 lists these with notes on their contributions). The remainder were recognized, to various degrees and in various forms, among reviews [2, 4, 16, 25, 29]. Other studies have identified negative factors this study did not; e.g., it did not identify power imbalances in the form of dependencies, which [4, 21] have noted relative to some genres of health partnerships.

The success factors identified have largely been recognized within the literature [2, 5, 13, 25–28, 30, 31]. However, some themes differed from literature in their emphasis on certain aspects. For instance, ‘Strategic vision and approach’ emphasized a compelling vision pursued systematically, while previous reports emphasized a shared vision [2, 13].

The occurrence of most negative factors with both struggling and potential partnerships suggests perceptions of potential partnerships’ prospects derive from circumstances and experiences broadly. This contrasts with previous reports’ emphasis on history with parties involved [19, 32].

This study reinforces calls for scholars to: examine struggling partnerships [2, 4, 17] and why potential partnerships go unexplored/undeveloped [4]; pursue cross-contextual studies transcending research siloes [17, 20]; and, assemble menus of frameworks and factors for practitioners to draw on when developing their own frameworks, as [33] demonstrated in another field. Partnerships’ complexity and practitioners’ unique circumstances imply researchers can often best help, as [12, 34] argue, by observing potential factors and reporting them non-prescriptively for practitioners’ consideration.

### Limitations

This study responded to limitations in the literature and itself had limitations. Its findings were neither comprehensive nor generalizable given its broad scope and qualitative exploratory design, and the sampling approach would not support claims to a representative sample. Nevertheless, the sample did incorporate a wide variety of participants and settings across the health sector. Inclusion of more or different participants and settings may have identified further or different negative and success factors, and may also have seen those mentioned in more contexts. Inclusion of two participants based in a closely related field instead of the health sector, while an exception, had no material implications. Their work had long included health initiatives with health groups, which they spoke to accordingly, and their comments did not distinctly affect any of the factors identified. This study used one interviewer and analyst (GZ), a potential source of bias; nevertheless, this supported continuity across interviews, analysis with the benefit of context, and deep immersion in the data. The cross-contextual heterogeneity of this study both supports and limits transferability. Negative and success factors identified may well apply elsewhere, to varying extent, partly because they were not counter-intuitive and many were broadly comparable to factors previously reported. This study included sources of potential selection bias: participants’ choices as to partnership examples they raised, and purposeful selection of those invited to participate. Participants’ choices yielded germane examples they were interested to discuss. Due to resource constraints this study excluded lower-income countries, a gap that merits future research.

## Conclusions

This study clearly suggests more attention be devoted to negative factors that contribute to struggling partnerships, including failed partnerships, and/or impede potential partnerships, including those unexplored and undeveloped. Scholarly attention to these partnerships and factors has been limited, especially for partnerships involving nonprofit partners in the health sector and more broadly. This study identified myriad negative factors and success factors: overall, these complemented each other. Almost all occurred across diverse contexts, including different regions and institutional sectors.

This study suggests three takeaways for practitioners. First, factors used to consider partnerships should reflect factors from struggling partnerships and/or potential partnerships, plus successful partnerships. Second, negative factors can highlight opportunities to help advance partnerships, individually and more systematically. Third, since practitioners face an abundance of potential factors, they should consider developing frameworks of factors from literature and experience to facilitate consideration of partnerships and inform approaches, lessons drawn, and potential partnerships sought.

## Supplementary Information

Supplementary Material 1.

Supplementary Material 2.

Supplementary Material 3.

Supplementary Material 4.

## Data Availability

The raw data underlying the current study are not publicly available due to them containing information that could compromise research participant anonymity and/or confidentiality. Reasonable requests for de-identified raw data will be considered by the corresponding author.

## Abbreviations

EU: European Union
EU+: Here refers to EU countries, Israel, Switzerland, United Kingdom
IOC: Inter-organizational collaboration
OECD: Organization for Economic Co-operation and Development
