# Supplementary material for: Vital lessons from struggling partnerships and potential partnerships: an international study with leaders across the health sector
Source: BMC Health Serv Res. 2024 Nov 26;24:1470. doi: 10.1186/s12913-024-11944-7 (PMC11590265; doi:10.1186/s12913-024-11944-7)
Supplement: Supplementary file 1 — Supplementary Material 1. [file 12913_2024_11944_MOESM1_ESM.docx]

**Additional file 1: Methodology** – includes interview guide

**Participants and setting:** Purposeful sampling (1) was employed to recruit a very wide range of leaders and managers involved in the health sector. The 70 participants—in 13 mainly OECD countries across Asia-Pacific, EU+, and North America—were recruited on a rolling basis over time to support representation of diverse settings, roles, and regions (Methods, Table 1), congruent with study aims (Background). Recruitment, by email/LinkedIn messages to individually selected invitees, drew especially from outside of author networks (n=52) with identification by internet searches (40/52) and selective use of snowballing referrals (12/52). Identification by internet searches drew on sources such as local health-sector institutions’ leadership directories and their press releases on various topics that mentioned key personnel involved, searches of local LinkedIn members in health-sector fields, and websites of health-sector associations. Recruitment also made selective use of authors’ networks (n=8) and referrals from authors’ networks (n=10).

Participants were recruited from a wide array of roles and areas across the health sector (e.g., clinical quality, health system leadership, pharmaceutical R&D, population health, patient advocacy and representation, policy advocacy, digital transformation) and were leaders and managers at different levels of seniority for organizations, units, teams, or initiatives in those areas (Methods, Table 1). All participants were based within the health sector except for two based in a closely related field, recruited from authors’ networks, whose work had long included health initiatives with health-sector organizations that they discussed accordingly in their interviews; these two participants thus represented a minor exception to the focus on participants based in the health sector. Participants worked in nonprofit, industry, and/or government settings, including research institutions, and were categorized as follows: (1) government participants worked for governments, including government-owned hospitals, or United Nations agencies; (2) nonprofit participants worked for nonprofits, including universities and associations of health professionals; (3) industry participants worked in the for-profit private sector. This study excluded individuals in sales and standard support departments or teams (e.g., human resources). Also, individuals were excluded if they indicated they could not speak to partnership topics in the interview guide sent in advance of interviews; in such cases, individuals emailed their regrets. Three participants worked for ≥2 organizations such that they had, for example, both nonprofit and industry affiliations (Methods, Table 1).

Recruitment and interviews were conducted in English from February to July 2017 (n=53), paused due to interviewer availability, and completed from August to November 2018 (n=17). The sampling approach does not support claims to a representative sample. Nevertheless, the sample did include participants from a wide range of health-sector settings.

**Terminology:** In the context of the present study, ‘partnerships’ and ‘collaborations’ are used interchangeably, and ‘struggling partnerships’ and ‘potential partnerships’ are used as defined in the Background section of the manuscript: those definitions are included here for convenience. ‘Struggling partnerships’ denotes partnerships agreed and embarked upon yet characterized by deficiencies/problems, including failed partnerships, partnerships representing close variations on failure, and those otherwise falling well short of their expected potential, in terms reflective of study participants’ descriptions (e.g., terminated, desultory, stymied, half-hearted, fraught, difficult, not working out). Meanwhile, ‘potential partnerships’ denotes all those, notional or otherwise not yet agreed and embarked upon, including: (1) those left unexplored in the first place, and therefore scarcely or not discussed with possible partners, termed ‘unexplored’; (2) those that had their exploration or development terminated, termed ‘abandoned’; (3) those otherwise undeveloped, termed ‘undeveloped’; and, (4) those under exploration or otherwise in development, termed ‘under exploration or development’.

**Interviews:** Semi-structured interviews were all conducted by GZ in accord with the aims of the study (Background), either face-to-face (n=22; 10 countries) or by phone (n=48) for 28-80 minutes (mean=52min, median=53min). Interviews were voluntary and confidential. With verbal consent, most interviews were audio-recorded (n=66), or detailed notes were taken (n=4). Afterwards, the interviewer transcribed audio-recorded interviews verbatim and thoroughly checked all transcripts. Participants were interviewed individually, except two interviewed jointly at their suggestion. Interviews were based on an interview guide which supported consistency across interviews while allowing flexibility to discuss and probe the partnership examples, factors, and related considerations raised by participants. Questions were not pre-tested and were revised after the second interview to enhance clarity and understanding. Recruitment and interviews were conducted in English.

Interviews focused on the elicitation and discussion of examples of partnerships and potential partnerships, corresponding to three categories of interest, and the negative factors or success factors involved, as described further below. A detailed interview guide to help orient participants, included at the end of this supplement, was: (a) shared with them in advance, usually 1-2 days prior to interviews; (b) reviewed with participants at the beginning of interviews to orient them to the aims, main topics, criteria for partnership examples, and confidentiality considerations; and, (c) referred to throughout. It was augmented with clarifying verbal guidance on certain topics, as described further below.

To help put participants at ease, and relatedly, to help minimize participants' potential concerns about whether to volunteer and discuss even potentially sensitive partnership examples and/or factors, they were: (a) assured of confidentiality; (b) informed examples and comments would be anonymized in any study report; (c) welcomed to disguise examples; (d) advised they were not expected to share sensitive information; and, (e) asked but not required to share examples of struggling partnerships, including failed partnerships, and advised they could instead choose to share alternative examples that were presumably less sensitive. The latter procedures are described further below.

In regard to how the study defined partnerships of interest, criteria applicable to all partnership examples were listed in the interview guide shared with participants (working title: “The Hidden Potential Study”) and reviewed with them at the beginning of interviews; the guide is included at the end of this supplement. The study was principally interested in inter-organizational partnerships (249/255 examples, the remainder are discussed below), as well as related partnerships, and the criteria reflected several considerations. Given the study’s exploratory nature, variety of participants, and desire to focus on partnerships of interest to participants, a key consideration was to avoid giving participants an overly restrictive sense of what organizational partnerships were of interest. Accordingly, the criteria allowed participants to talk about formal and/or informal partnerships and collaborations, used here interchangeably, including: (a) inter-organizational collaborations; (b) collaborations of leaders or groups (i.e., leaders/experts who might collaborate over time even as their organizational affiliations change; a few were discussed, within examples of inter-organizational collaborations); (c) collaborations involving various organizations and internal units; or, (d) only internal units (these intra-organizational partnerships accounted for the remainder of examples, 6/255; all 6 were set within large or very large organizations). The options with internal units were given for two reasons: firstly, because internal partnerships in large organizations, such as health systems, can resemble inter-organizational partnerships; and, secondly, to help participants grasp the study's interest in inter-organizational partnerships extended to internal collaboration those involved.

The remaining criteria applicable to all partnership examples were: (a) not simply a transaction or vendor-client relationship; (b) relevant to participants' current role or, for examples from their past, to their role then; (c) relatively distinctive from the participant’s perspective; and, (d) health-related, i.e. supportive of better health outcomes and ideally access. Many endeavors in the health sector support health outcomes, whether more directly as with healthcare or less directly as with pharmaceutical research. The health-related criterion served to exclude examples concerned only with commercial goals or otherwise unrelated to health. Mergers were excluded, in accord with criterion (a) above.

Participants were asked to volunteer and discuss three partnership examples, one from each of three categories: (1) successful partnerships; (2) struggling partnerships; and, (3) potential partnerships. The approach and questions employed for each category follow.

The first category was successful partnerships. Participants were asked to discuss a successful partnership they felt enthusiastic about, why, and what was essential to achieve it (i.e., success factors).

The second category was struggling partnerships, including failed partnerships, that participants considered otherwise worthwhile in principle. The interview guide shared with participants intentionally featured a more ambiguous term, ‘underutilized partnerships’. This aimed to help elicit examples of partnerships participants considered remarkable in the first instance for their seeming potential rather than just for their dysfunction. Early in the interview, the interviewer clarified that the category of principal interest was struggling partnerships, while also offering the option to instead discuss an alternative category. The interviewer did so by telling participants: (a) they could go different ways with their choice of what example to share; (b) the question was mainly about struggling partnerships with otherwise seemingly remarkable promise, yet falling well short of their expected potential; (c) most participants so far had given examples of struggling partnerships, including failed partnerships; (d) alternatively, they were welcome to raise a different example, such as a partnership that worked well and could exploit its greater potential. Notably, this approach enabled participants to opt-in to raising examples of struggling partnerships, so they might feel more comfortable and inclined to share informative examples from this often sensitive category. Further to their examples of struggling partnerships, participants were asked about the partnership’s original promise, its current status, and contributing key barriers/challenges (i.e., negative factors).

The third category was potential partnerships that participants considered worthwhile, or at least otherwise worthwhile in principle. To elicit examples of potential partnerships, participants were asked to discuss the following: a ‘dream partnership’ they would very much like to have or see, irrespective of prevailing constraints, and why; key barriers/challenges (i.e., negative factors) impeding it; and, what might be essential to achieve it. Notably, this approach enabled participants to raise examples of unexplored and/or undeveloped potential partnerships, as well as other examples of potential partnerships, such as potential partnerships under exploration or development.

Many participants volunteered and discussed somewhat more than three relevant examples in total (mean=3.7, median=4) as they were not restricted to discuss only one example for each of the aforementioned categories. The interviewer probed for understanding of: struggling partnerships, potential partnerships, and negative factors involved in each; and, successful partnerships and success factors involved in those.

Interviews included up to two further questions. The penultimate question, covered only in case of extra time, asked what partnerships the participant wished they and/or their colleagues had greater capacity to identify and develop. This sometimes prompted participants to raise additional, germane examples of potential partnerships or to expand on those they had just raised. These were discussed and negative factors probed as time allowed. Such examples were later analyzed normally except comments were not coded to the theme 'Interested and suitable partners hard to identify, not aware of each other' as the question might be considered leading in this vein. The final question inquired as to any questions or suggestions the participant might like to venture.

**Data analysis:** After all data collection was complete, interview data were analyzed thematically by GZ employing principles of thematic network analysis (2) following the approach taken in the study by Aveling and colleagues (3) as discussed further here. This approach was attractive for several reasons, including its flexible support for: (1) development of an organized set of themes, or thematic network, around a global theme matching a research/interview question; (2) a degree of interpretation and data reduction appropriate to this study’s aims and themes’ accessibility to practitioners; (3) use of tables to illustrate each set of themes; and, (4) the iterative analytic process involving multiple passes through the data. Although they (3) employed deductive-inductive analysis while the present study used inductive analysis, this difference did not indicate incompatibility with their approach otherwise. Thematic network analysis is suitable for inductive, deductive, or combined analyses (2).

The analysis aimed to address the primary research questions, namely: among a broad range of inter‑organizational and related partnerships, and potential partnerships, each raised as worthwhile or otherwise worthwhile in principle by interviewed leaders and managers (i.e., practitioners) drawn from 13 mainly OECD countries across different regions and diverse roles across a wide array of nonprofit, government, and industry settings in the health sector, and based on factors they mention in relation to their individual examples, (1) what negative factors contribute to struggling partnerships and/or impede potential partnerships, and (2) what success factors contribute to successful partnerships?

Accordingly, separate thematic networks were developed that centered, respectively, on two global themes connected to the research questions: (1) negative factors that contribute to struggling partnerships and/or impede potential partnerships, and (2) success factors that contribute to successful partnerships. Analysis for each global theme involved iteratively deriving and refining a set of themes, grouped under organizing themes, and applying this coding framework across all transcripts. This analytic process involved multiple passes through the data at each stage from initial familiarization to application of the final coding framework. Theme identification used inductive coding. Analysis was supported by Nvivo software (Lumivero, Denver, CO) and did not use auto-coding. Throughout, constant comparisons were made within and between interview transcripts to seek contrasting views and deviant cases so as to further inform and refine the themes under development (no contrasting themes or deviant cases emerged). To support this study’s bottom-up and inductive analytical approach, previously reported frameworks and collections of negative factors, success factors, and related considerations were not consulted during data collection or the thematic network analysis.

Themes were based on participants' comments and reflect those comments' commonalities and variety (see also Results, Tables 2 and 3, as well as Additional files 2 and 3). For example, the theme 'Negotiations, alignment building, contracting involved seen as: difficult, prolonged, vulnerable to failure, too time and resource intensive' derived from comments to the effect either the negotiations, alignment building, contracting, or some combination thereof involved in the partnerships or potential partnerships in question were variously seen as either difficult, prolonged, vulnerable to failure, too time and resource intensive, or some combination thereof.

The analysis also aimed to address secondary research questions, namely: (1) for all negative factors and success factors identified as themes, what range of institutional sectors (e.g., nonprofit, industry) and international regions (e.g., Asia-Pacific) in the study did each represent, regarding the participants whose comments they reflected; and, (2) for each of the negative factors themes identified, did the underlying comments concern struggling partnerships, potential partnerships, or a mix of both? For this analysis, after all themes were finalized, the participant comments coded to each theme were reviewed. This review noted the participants' regions (Asia-Pacific, EU+, or North America) and institutional sectors (nonprofit, government, or industry), specifically any regions and institutional sectors not represented, as well as whether participants were concentrated in one area/field (with reference to any of the ‘areas of focus and leadership’ listed in Table 1, e.g., ‘health services delivery systems, operations, innovation and improvement’, ‘global health’, ‘hospitals’, ‘pharmaceutical R&D’, ‘patient voice and involvement’). Three participants worked for ≥2 organizations such that they had, for example, both nonprofit and industry affiliations (Methods, Table 1). In the case of any theme in which their comments were among the underlying comments, the review considered their role, organizational and instutional-sector affiliation relating to the specific partnership example their comments concerned. For negative factors, all underlying comments were also reviewed, for each theme, as to whether they had been made only in discussions of struggling partnerships examples or of potential partnerships examples. These reviews were intended to assess basic indications of each theme’s cross-contextual range, within the scope of this exploratory qualitative study.

**Ethical considerations:** The Seattle University institutional review board (IRB) found this study exempt from the need for IRB review (letter dated February 2, 2017). All methods in this study were carried out in accordance with the declaration of Helsinki. Interviews were voluntary and confidential. With verbal informed consent, interviews were audio-recorded for transcription (n=66), or detailed notes were taken (n=4). Participant information and quotes have been anonymized and disguised.

**Interview guide:** This is provided further below, as well as the version shared with participants.

**Note on rights and permissions:** The original authors of this document (“Additional file 1: Methodology”) are Greg Zwisler, Christopher Sauer, and David Shoultz. The original source, for citation purposes, is their manuscript entitled “Vital lessons from struggling partnerships and potential partnerships: an international study with leaders across the health sector”, published by BMC Health Services Research. This Additional file and its contents are licensed under a Creative Commons Attribution 4.0 International License, which permits use, sharing, adaptation, distribution and reproduction in any medium or format, as long as you give appropriate credit to the original author(s) and the source, provide a link to the Creative Commons licence, and indicate if changes were made. To view a copy of this licence, visit <http://creativecommons.org/licenses/by/4.0/>.

References

1. Moser A, Korstjens I. Series: Practical guidance to qualitative research. Part 3: Sampling, data collection and analysis. Vol. 24, European Journal of General Practice. 2018.

2. Attride-Stirling J. Thematic networks: An analytic tool for qualitative research. Qualitative Research. 2001;1(3).

3. Aveling EL, Stone J, Sundt T, Wright C, Gino F, Singer S. Factors Influencing Team Behaviors in Surgery: A Qualitative Study to Inform Teamwork Interventions. Annals of Thoracic Surgery. 2018;106(1).

**Interview guide shared with participants**

*See also the data collection section of methodology above and the interview guide on next page.*


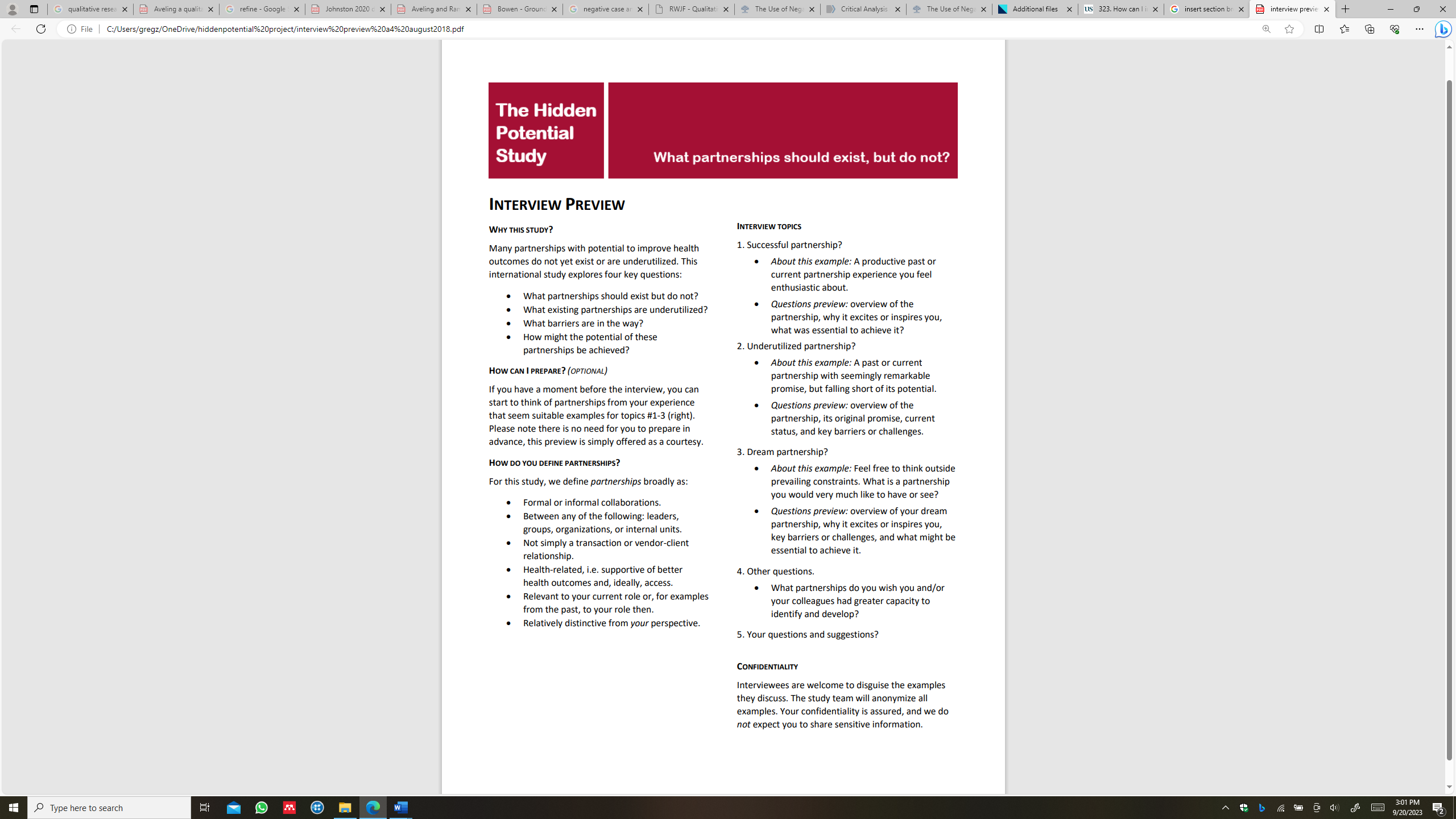


**Interview guide** – for use alongside version shared with participants

*See also interview guide shared with participants and the data collection section of methodology, above.*

**Orientation and other preliminaries**

Hello! Thank you very much for your willingness to participate in this study. I will go over the interview preview and a few other things with you before we get into the interview. May we begin with quick introductions? *Give professional self-introduction, participant introduces themselves, their role, etc.*

Thank you. I will go over the interview preview that I sent earlier, do you have it there with you? *Confirm.*

Great, please jump in anytime with any questions you have as we go over it. First, why this study? Many partnerships with potential to improve health outcomes do not yet exist or are underutilized. This international study explores several key questions including:

- What partnerships should exist but do not?
- What existing partnerships are underutilized?
- What key barriers or challenges get in the way?

So, how are we defining partnerships? For this study, we define partnerships broadly as:

- Formal or informal collaborations
- Between any of the following: leaders, groups, organizations, or internal units.
- Not simply a transaction or vendor-client relationship.
- Health-related, i.e. supportive of better health-outcomes and, ideally, access.
- Relevant to your current role or, for examples from the past, to your role then.
- Relatively distinctive from your perspective.

The interview topics are listed in the interview preview, on the right side.

We’ll focus on discussing each of 3 partnership examples you share from your perspective—successful, underutilized, and dream partnerships—and for each, key factors. These examples are labeled #1 to #3:

- A successful partnership—a productive past or current experience you feel enthusiastic about.
- An underutilized partnership—this example is mainly about struggling partnerships including failed partnerships, but you will be welcome to raise a different kind of example if you like.
- A dream partnership—one you would very much like to have or see, feeling free to think outside prevailing constraints.

Your confidentiality and privacy are very important to us.

You are welcome to disguise the examples you discuss. The study team will anonymize all examples. Your confidentiality is assured, and we do not expect you to share sensitive information. Your participation in the study is entirely voluntary. You may stop the interview anytime or refuse any of the questions. We are scheduled to talk for around 45 minutes, does this conversation still fit well in your schedule today?

I would also like to ask your permission to audio record this conversation. This helps me to capture and transcribe everything we discuss. We will not share the recording outside of our research team. In any study reports and presentations, we will anonymize all examples given and statements made, and will not include your name. If you prefer not to be recorded, I can just take notes instead. Would you feel comfortable to be audio recorded for this interview? *(if they answer yes, turn on recorder)*

**Interview topics** – with interview questions

**Topic 1. Successful partnership?**

The first partnership example we’ll discuss is one you consider a successful partnership—a productive past or current partnership experience you feel enthusiastic about.

Please let me know if you have any questions, think of an example you would like to give, take your time.

Please give an overview of the partnership and tell me why it excites or inspires you.

*Probe for understanding, note the range of organizations involved and their institutional sectors*

What was essential to achieve it? *(success factors)*

*Probe for understanding the factors they raise*

**Topic 2. Underutilized partnership?**

The next example we’ll discuss is an underutilized partnership—a past or current partnership with seemingly remarkable promise, but falling short of its potential. Please note you can go different ways with your choice of what example to share. This example is mainly about struggling partnerships, with otherwise seemingly remarkable promise yet falling well short of their expected potential. Most participants so far have given examples of struggling partnerships including failed partnerships. You are welcome to raise a different example, such as a partnership that worked very well and could exploit its now greater potential, if you would rather do so instead.

Please let me know if you have any questions, think of an example you would like to give, take your time.

Please give an overview of the partnership, its original promise and current status.

*Probe for understanding, note the range of organizations involved and their institutional sectors*

What do you see as its key barriers or challenges? *(factors contributing to its difficulties/problems)*

*Probe for understanding the factors they raise*

**Topic 3.** **Dream partnership?**

The next partnership example we’ll discuss is one you consider a dream partnership—feel free to think outside prevailing constraints—what is a partnership you would very much like to have or see?

Please let me know if you have any questions, think of an example you would like to give, take your time.

Please give an overview of your dream partnership, and why it excites or inspires you.

*Probe for understanding, note the range of organizations involved and their institutional sectors*

What do you see as the key barriers or challenges to it? *(factors impeding or even preventing it)*

*Probe for understanding the factors they raise*

What might be essential to achieve it? *(may prompt other key barriers or challenges, probe any raised)*

*Probe for understanding*

**4. Other questions.** *(ask if time allows)*

What partnerships do you wish you and/or your colleagues had greater capacity to identify and develop?

**5. Your questions and suggestions?** *(ask)*
