# Supplementary material for: Vital lessons from struggling partnerships and potential partnerships: an international study with leaders across the health sector
Source: BMC Health Serv Res. 2024 Nov 26;24:1470. doi: 10.1186/s12913-024-11944-7 (PMC11590265; doi:10.1186/s12913-024-11944-7)
Supplement: Supplementary file 2 — Supplementary Material 2. [file 12913_2024_11944_MOESM2_ESM.docx]

**Additional file 2: Negative factors and illustrative quotes**

Listed in the same order as in the manuscript (Results, Table 2). Illustrative quotes are anonymized and disguised.

| **Organizing theme**  **Problematic structures and mentalities within the health sector** |  |
| --- | --- |
| Theme  Health finance structures, the ways money flows, their flaws and effects  e.g.,  - the workings of health finance foster problems in terms of unproductive incentives, disjointed activity, obstacles to useful changes and initiatives  - embed dysfunction, other negative factors (see also theme ‘misaligned underlying interests, aims, incentives of those involved’)  - insufficient allocations, to prevention relative to treatment | Illustrative quotes  “The other barrier is obviously money, it’s the financing structure. I think sometimes the flow of the money doesn’t follow the desired flow of care. Oftentimes it will start with good intentions – say let me give you a blended rate to take care of certain populations which includes a certain mixture of simple, intermediate, and complex cases. The problem is everyone tries to optimize the funding – they tend to gravitate to the simple cases.” {223, government, Asia‑Pacific}  “And then you need the care delivery system designed to perform with that benefits design system in that network with those population health approaches – oh that’s good, but then the doctors are paid to do X-rays and MRIs even though the health system is paid not to. I mean Medicare is a perfect example they pay the hospital a DRG, a case rate to the hospital. How do they pay the doctor? They pay them an RVU per day. So right there Medicare says hospital incentivized to reduce length of stay – what’s the doctor incentivized? Bring ‘em in and keep ‘em in. […] The reimbursement often isn’t aligned.” {233, industry, North America}  “Part of [the challenge] is about the financing of the global health landscape and the ways competition are encouraged, the emphasis perhaps on more proximal outputs of projects without an emphasis or incentivization specifically for collaboration […] and the need to plan to meet certain targets that are required through external funding mechanisms and the way that drives behavior […] Those resources could go farther to address more health needs if they were allowed to be sort of jointly shared or at least co-planned…” {262, government, EU+}  “People are very protectionist of territory, and I would say healthcare is not free from that because the way we’re all funded is, ‘This is your money, this is your money, this is your money…” {240, nonprofit, North America}  “..to us [this area of health] is a lot about awareness about prevention about seeking help sooner, so if we just treat people when they are already suffering they are already acute. We always feel our work should be more [about prevention in the community] but because of the funding model, because of the current work and our constraints we are unable to do that…” {238, government, Asia‑Pacific} |
| Theme  Prevailing mental models and general inertia of: health systems, health policy, medical culture, routine clinical delivery  e.g.,  - familiar mental models, work of health systems and healthcare, and how accustomed people are to these, together foster inertia, preclude or limit ideas, change  - medical culture, its hierarchies, roots in familiar ways, training  - health policy, its inertia limits change  - clinicians’ accustomed ways of working and related beliefs | Illustrative quotes  “The other barrier would be there is a challenge to find the common mental model, what we want to achieve. We’re talking about [more] primary healthcare less hospital care, but really to define this together would be [very hard]. The providers are trained in the system, they have gone to the medical schools and the nurses’ schools, they have worked in the system so there’s an established mental model. Even if you can change the structure this will need a change of culture which is a much harder thing to do […] it’s hard to think and to see something that you haven’t tried.” {239, government, EU+}  “There’s a lot of inertia that comes out of that deeply held mindset [of the administrative treatment of healthcare] all of the apparatus that grows from it – that can be the source of people’s professional prestige the way that money gets allocated, problems get framed that way, research all kind of organizes itself around that stuff. It’s premised on a narrow kind of problem framing…”  {229, nonprofit, North America}  “You have a strong medical culture you also have policies made on different levels in healthcare you have international policies national policies […] and then you have medical culture and then on the working level – on the wards – you have specific departmental culture or policies, ‘This is how we do things here’ […] When that staff from bottom up [propose change initiatives] they will collide with the policies and the culture that comes from the medical system and top down instead, so the change they want to do they will not be able to do…” {227, government, EU+}  “..with all the senior clinical leads we also struck the fact they truly believed [the only way] patients would have the best care was if [patients] came and saw [them] so that also became you think of change management and it’s not just resistance to change it’s also a strong belief that what they do currently is in the best interest of the patient, ‘It’s not just that I don’t want to change, it’s also that I’ve invested my whole career and my clinical hierarchical position, that this way of treatment is the best.” {255, industry, EU+} |
| Theme  Extensive rules and regulations  e.g.,  - burden, preclude or constrain the partnerships, their projects  - for pharmaceutical industry, health insurance, healthcare, other areas in health sector  - to do with patient data, privacy, research  - inflexible, broadly applied | Illustrative quotes  “..that kind of rule prohibits lots of potential interesting partnerships you could do with the payers like a patient monitoring program, something you can add on the pill to help your partner to achieve better health outcomes, but because the regulator is afraid you may misuse this additional value offering to gain unfair market access or to influence their decisions so that is ruled out. […] There are reasons for it. Pharma has done a lot of wrong things in different places. But then the rules are created in a way to weed everything out – they are little bit too sensitive and not specific enough.” (n.b., ‘sensitive’ and ‘specific’ are used in their senses as technical terms for performance of diagnostic/screening tests, here in an analogy to refine the broad point made) {219, industry, EU+}  “..but in [these government-funded insurance] plans there are very structured regulations about what are permissible value-added benefits and ways to go about [testing new things], so that’s complicated.” {253, industry, North America}  “Another barrier is the [patient] privacy law. Some data are prohibited to use or to analyze by this law, erroneously understood, too widely applied…” {201, nonprofit, Asia‑Pacific}  “The big roadblock we are facing actually is you have the government which has suddenly changed the rules as in providers [like us] need to fulfill certain criteria […] and if we don’t we have to fold the [joint] program. So that's why we end up running around in circles looking at how we can fulfill the criteria […] That you have to do, you can't quite be creative about it because if they say that for every two [such and such] you can only have one [of that] that's a very it's set in stone, you have to do it […] We’re getting consultants in to take a look and see because it’s a lot of paperwork, the amount of paperwork is insane…” {249, nonprofit, Asia‑Pacific} |
| Theme  Paternalism, traditional mindsets limit meaningful patient involvement  e.g.,  - failure, difficulty to substantially reorient vis-à-vis patients generally, and engage their priorities/involvement, as more traditional mindsets still continue to be prevalent in healthcare, patients seen more as passive recipients of care  - limiting views of patients with stigmatized conditions, their capacity and suitable care  - skepticism, limited appreciation of the value of patients’ insights, input on relevant topics, issues beyond their own health matters | Illustrative quotes  “..there is still a very paternalistic sentiment within the healthcare industry – private, for-profit, not-for-profit, public, it is across the board. […] We pay a lot of lip service, we talk about patient engagement about patient oriented outcomes – it’s still not meaningful, it’s very figurehead in my opinion.” {236, government, North America}  “..the barrier is every education system around the world still training folks to be provider centric, not [oriented to what’s most important to the patient]. You’re trained to be a clinical expert and then you go to an organization that wants you to drive their mandate. […] People are just trained with their lens. There’s no shame and blame here – it just requires a different orientation and a different way to look at being in a caring field.”  {237, nonprofit, North America}  “We felt our patients are stigmatized even by the healthcare workers in the [partner healthcare organizations]. So the stigma is in a different way in this kind of [facility] they will think, ‘Okay, since you have [a stigmatized health problem] whatever you’re saying may not be true.’ They just don’t take our patients very seriously when they come in about [other health problems] and symptoms. They don’t listen very well when it comes to our patients and sometimes they also assume that because you are suffering from [a stigmatized health problem] therefore you don’t have the capacity to make decisions – so they don’t really think they need to get patients’ agreement to the care plan.” {238, government, Asia‑Pacific}  “..a mindset which is, there’s no other way to say this other than the old school, ‘This is disability and there’s no capacity and no ability,’ that mindset of, ‘We need to protect these people, we need to treat them as a charity.’ I think our big challenge is changing that perception to one of strengths and capacity. These are not people who need to be mollycoddled and looked after […] I think old school views tend to be blockers…”  {260, nonprofit, Asia-Pacific}  “We also have [here in the capital city] people who used to be the [main] ones who were consulted – originally we worked with the professionals, the scientists, the workforce administration – and for them a new stakeholder coming is not necessarily easy. But [the patient representatives] have a knowledge we do not have because they have the user perspective. To admit this knowledge is at least as important as the science is not something easy [for the original stakeholders]…” {228, government, EU+} |
| Theme  Insularity, not going outside health sector or home country to learn from, work with others  e.g.,  - insularity of the health sector  - not much inclined to go outside, engage more across boundaries  - in other industries, social sectors  - limited supply of personnel with broader interests, experience | Illustrative quotes  “Healthcare tends to be a very insular world, I am in healthcare so I can say that. […] Everywhere I go there are the same problems that are facing healthcare with some jurisdictions already over the precipice and yet […] when I speak to my colleagues about the different partnerships or innovations going on in [country A or B] or even in [country C] where I have contacts there’s always this thing of, ‘You know there’s not really much we can learn from [country B] because it’s privatized,’ or, ‘There isn’t much we can learn from the [health system in country A] look at how broken it is, blah blah blah.” {236, government, North America}  “One thing I’ve been surprised at are the people who don’t know each other […] I mean obviously the healthcare people know the insurance people but they don’t necessarily know the banks, or they have linkages [to different industries] but they haven’t take advantage of them yet, they haven’t really sought it out.” {206, nonprofit, Asia‑Pacific}  “..when the health people got together their response was, ‘Well, maybe we don’t have to care about education and economics because other tables are doing those things’ […] so conceptually they [eventually] began to see that ‘We as health leaders should speak up and champion these other groups and ensure they are successful and fully funded – but operationally we don’t have to work together because these other groups have formed,’ and that was kind of a – it was a long slog to get there months of fits and starts and trying to understand to move off this position of ‘[Operationally] health is going to deal with things that only the health people are going to address’ and that’s kind of a tragic view of what partnerships can do […] it's based on narrowly framed separate disjointed I mean another way to put it, it’s like wholly guided by the concept of parallel play…” {229, nonprofit, North America}  “This should be one of the rules of accreditation [for medical institutions] how many [international] cooperation you have with others […] to communicate, to compare, to promote and with other – look do you know what are the medical [policies] in Scandinavia let us go to Scandinavia learn it and take the good things but you cannot look once and it’s finished it needs to be continuous and connected, if you put this in the accreditation you will push the institutions. […In terms of barriers…] we have not enough people to think medicine and organization and also you will very rarely find physicians who are involved with that and who like it. I think that this is extremely important but I am not sure that we are many who think about these things as important issues. A lot of physicians – of my friends – do not care on the [broader] view thing. We are limited to think about the patient we see everything through the patient but not through the crowds and I think you have international justice, international laws, and these international lawyers are very in demand – we need more of these type in medicine – [international] affairs medicine!” {212, government, EU+} |
| **Organizing theme**  **Misaligned motivations and competitive dynamics** |  |
| Theme  Misaligned underlying interests, aims, incentives of those involved,  e.g.,  - of partners, organizations, sub-units, individuals, groups  - their own interests, broader objectives, organizational goals either conflict or are otherwise not aligned  - their incentives interfere with collaboration, limit its scope (see also theme ‘competition, partners’ needs to prioritize their own competitiveness’) | Illustrative quotes  “I think the professional organizations they will – it’s hard for them to discuss to make task shifts – they have to lose some power they would have to let other professionals do some of the work done by nurses or doctors, that would be one very big problem. […] There may be some differences in interests among different groups – because it’s about power.” {239, government, EU+}  “It was a large group with lots of partners and interests were not aligned, we had varying interests.” {270, government, EU+}  “We had very different [products and] business models […] so there were challenges with that partnership because of the wildly misaligned incentives […] You may [also] spend a lot of time [together] trying to develop [a custom accessory for a medical product] that doesn’t really need it just because there’s a corporate goal that maybe people’s bonuses are attached to so maybe people are trying to optimize their bonus as opposed to optimize really what’s going to be best for the business...” {225, industry, North America}  “There’s a book from Seth Rodin, ‘This Is Broken,’ the first one is, ‘Not my job’ – and I think between the different [public] agencies there is kind of that ‘I am a jurist and it’s my job to make sure the law is interpreted in the harshest way, and it’s not my job if that makes people unemployed’…” (i.e., by effectively limiting other agencies’ initiatives to develop the health sector and economy) {265, government, EU+}  “Whether you send the patient to me shouldn’t be a matter of [your bed occupancy] statistics it should depend on whether the patient would be better served […] but we all have our own self-survival instinct. If you are running your hospital you want to make sure your numbers look good – otherwise the [government] will send [someone to replace you]. You shouldn’t be penalized for sending the right people to [us]…” {243, nonprofit, Asia‑Pacific} |
| Theme  Competition, partners’ needs to prioritize their own competitiveness,  e.g.,  - competitive dynamics, as with competing companies, nonprofits  - constrains information sharing, work together, pursuit of broader/shared interests  - misalignment on account of competition and incentives to compete (see also theme ‘misaligned underlying interests, aims, incentives of those involved’)  - partners reliant on fundraising, such as NGOs and those in academia, must keep their competitiveness front of mind and so are wary to dilute their visibility, competitive position, etc.—these dynamics can attenuate partnerships involving groups that compete or simply need to focus their efforts and resources more on their own initiatives, objectives | Illustrative quotes  “I wish we could do a little more in partnership with our competitors, to be honest. Now that’s a little bit dicey because we’re competing for market share and all that kind of stuff but you know [in this newer area of our field] they are facing the same issues that we are and they have a tremendous amount of knowledge just like we do. […] It’s all the [barriers] you would suspect, there’s certain competitive advantages we’d maybe figure something else out, we don’t necessarily want to show all our cards.” {253, industry, North America}  “I think one of the limiting factors in [that country] for these types of institutions to work together is the competition there is. Specifically, each one is saying in a way that ‘We want to be the best,’ there’s competition that goes on the patient side and trying to draw more patients to your hospital, but also from a grant perspective as you try to pitch whatever you’re doing whether it’s innovation or clinical medicine you’re leading the country in that.” {222, government, EU+}  “That [partnership] is something all the universities say we needed but it’s underutilized because with the competition you have in science and each of the institutions – [institutions A, B, C, D, E] – they still have to survive as an institution and the survival in a network – of a [national] network is not the prime thing where they get the funds…” {217, nonprofit, EU+}  “It was pretty clear that [the nonprofits] felt each of them had the mandate to deliver the [redundant] technical products that they were delivering […] and the incentive to harmonize align and reduce duplication is actually not that present in the way that global health structures are financed and the way that additional financing comes to organizations right now – and so we were sort of working against the incentives that people have to make their work more visible and more prominent in the global health landscape, and I think as people very rightly perceive that the investments are decreasing there was a lot of anxiety about what not having [their own logo] be the primary label on the product, not being able to demonstrate individual expertise or individual accomplishment [of their] organization might mean.” {262, government, EU+} |
| **Organizing theme**  **Deficiencies in leadership, partnership management, and communication** |  |
| Theme  Lack of strong leadership  e.g.,  - relative to the partnership, an absence of leaders and leadership with considerable initiative, vision, skill, conviction, engagement and interest, courage to take risks  - to effectively address needs and circumstances involved  - among people involved, by senior organizational/elected leaders as to the partnership  - in connection with aspects of the institutional environment, as when attributed to culture | Illustrative quotes  “[Originally] it was working well – these were [several] organizations and there was a clear leadership, very very clear, from [an individual at] one of the organizations and the others followed. […] But the leadership and the strategy making was not incorporated in this partnership so [when] the person left a huge hole was there. […] So, two things happened there – on one hand the strong leader was missing, on the other hand [the representatives of the various partners] were changing too much…” {245, nonprofit, EU+}  “..many people [were] excited and had no issues with it but the leadership in that group has been born and raised in the bureaucracy, so I really started to appreciate how this place is wired and any bureaucracy would be. […] I think what I’ve encountered is that I’m dealing with a lot of managers versus leaders – you know the whole notion of leaders take risk, they take chances, they try new things, they get on the frontlines to empower them to do what they think they should do.” {232, government, North America}  “..actually it’s very hard to keep [such partnerships] on track. It requires very strong leadership and very tight agreement between [the organizations] on what their strategies are to keep on track and I think the partnerships lack that kind of management very often and then they start to sort of fray at the edges or big projects just don’t come into the partnership so it just becomes hot air. There’s very few examples I see around the world where these strategic partnerships have truly developed something really strong that lasted for years between the two [partners] you can count on one hand the number of [organizations] that have done it [and in those cases] they will really insist over time on many face-to-face meetings of the top management, really the top management the actual decision makers of the company and [institution] get together and they have several meetings and said are we still aligned? […] I think in many of the partnerships that fail that doesn’t happen.” {257, nonprofit, EU+}  “We’ve had some big things introduced [in the past]. But it takes conviction and it takes a leader who’s prepared to take a bit of a crash or crash through approach really.” {264, nonprofit, Asia‑Pacific}  “I think particularly the first time you talk, three or four partners they get together they always say, ‘Yes we have to do something,’ but different region different culture for example in Asia there is lack of leadership, leadership also need to take the responsibility not only need to design the project also need to make efforts on the funding […] In Asia culture it is the lack of leadership, this I think a lot I observe myself. {204, government, Asia‑Pacific} |
| Theme  Lack of partnership skills and experience  e.g.,  - those involved lack relevant experience with partnerships  - suitable, experienced people in short supply in organization, or more broadly in setting  - lack of adequate skills, in individuals or more broadly, for partnerships, collaboration  - lack of training, as broad challenge in sector | Illustrative quotes  “I guess [part of the problem] comes from the very different backgrounds of the different companies [many] have a team of founders that’s very dominated by real tech people, not tech people and developers who have done a lot of industry projects before but rather like tech people who have been deeply involved in their line of [biomedical] research and have no real exposure to business topics – many do come from a medical background, of course they have not had the previous exposure of business or business partnerships.” {224, industry, EU+}  “[The negative factor is too few of our researchers have] international cooperation capacity. Someone who is quite good, those good ones every two or three years they go to other countries to study for maybe half a year – then they get more rich experience, then they are interested to involve in this kind of activities and their capacity become more and more rich.”  {204, government, Asia-Pacific}  “..like everything in healthcare, [partnerships is] actually a craft, it’s a learned skill, and where would you learn this stuff? Like, there’s no training. […] I think that is part of why it is not done at all or well is people actually don’t know how to do it. They’re not trained.” {216, nonprofit, EU+}  “..from what I’ve seen coming to companies’ success or not success [in these collaborations with our institution] I think it’s extremely important who they send to work with [us]. It shouldn’t be but it’s a lot about social skills […] you need to be, like the company you’re coming from all those questions coming from there all those demands as a company and it’s the same from [our] hospital so you need to make people feel very comfortable.” {258, government, EU+}  “Another [negative factor] is that people don’t have the skills yet to collaborate – I mean really collaborate.” {237, nonprofit, North America} |
| Theme  Deficiencies in communication practices, mechanisms, skills  e.g.,  - inadequate meeting modes or frequencies, unhelpful use of lingo, other poor practices  - missing mechanisms for dialogue in or between varied and broad groups  - lack of skills and awareness to navigate differences in language, lingo  - to engage with those from different sectors (see also theme ‘hard for industry and government partners to engage, work together’) | Illustrative quotes  “One of the things from my perspective and my experience is communication, because when you have communication things happen. […] What I usually try to initiate is to have conversations very much like working with [a quite successful partnership] where we set a time to communicate on a webinar whether it’s once a month or once every two months. […] But then with [our partner in this underutilized partnership] when they come over to [this country] they jump in here, we spend a day together and we discuss why nothing much is working – so that's I think one indication where you don’t have those open communication channels.” {222, government, EU+}  “The biggest challenge is actually a quite simple one – when you really want to innovate [at the technical level] and create something new on that sort of [European] scale and level you really need to get some people really together. The risk is you end up having all sort of people in different places who meet in Skype calls – and my feeling about Skype calls is you use like ten per cent of the intellectual capacity which is available and you don’t get anywhere, especially not that fast.” {250, industry, EU+}  “..that’s really difficult because there’s no formalized structure of collective dialogue. So we have individual partnerships on very singular issues but it’s very difficult to bring that, ‘Here’s the latest thing, here’s research, here’s the innovation agenda,’ in a collective way [with the region’s many local governments].” {209, government, EU+}  “We’ve had so many companies coming to us speaking the corporate lingo and almost driving physicians away – everything they’re saying is fine but the language which they’re using is wrong so antagonizing people making them go in the opposite direction – and if you don’t take that into account and factor for it then you’re not going to go anywhere. We try to be the interpreters but it would be nice if we didn’t have to all the time […] and I would say that the problem could be even bigger in [our country] because we’re publicly funded so physicians don’t need to talk the same about money, of course they need to talk about keeping the budget but it’s not the same…” {259, government, EU+} |
| Theme  Objectives, activities insufficiently focused, not clearly defined  e.g.,  - objectives unclear, unspecific, not readily or fully understood  - activities unfocused, too broad  - in the beginning, throughout  - not meaningful enough overall, pursuing too much | Illustrative quotes  “The same reasons that led [the partnership I described earlier] to be successful are the same ones that led this one to be unsuccessful. So, for example the lack of definition of clear deliverables and clear timelines. There was a bit of a sense of mission creep – adding things on top that weren’t originally there, kind of like losing focus and getting sidetracked. […] There was an opportunity to do really good things but the lack of clear definition of objectives in the beginning was a major flaw.” {246, industry, EU+}  “Because in the beginning they don’t fix the real objectives.” {221, industry, EU+}  “Maybe the less clear dimension of the goals. The [other and more successful initiative] worked when it was ‘[Intervention name] for children’—it has to be very specific...” {213, industry, EU+}  “..I can’t tell you how many meetings for these various partnerships I go to where it’s the same representatives from the various organizations at all of these meetings. So I think part of it is almost over-coordination, that […] actually [they have] no time to deliver on the mandate of [one] partnership because they’re trying to manage all the other partnerships that they’re also a part of! I think that challenge is something we need to think critically about and it might mean that the global health world needs to stop doing some things in order to start doing some things that might be more meaningful.” {262, government, EU+} |
| Theme  Not adequately understanding partners, their needs  e.g.,  - limited grasp of how different partners or kinds of partners work, their perspectives, where they are coming from  - not working effectively to understand partners, needs  - poor sense of partners’ relevant needs, realities, cultural/sectoral contexts | Illustrative quotes  “..I knew nothing about the [corporate partner], about the size of the budgets […] I was pretty much an academic who had come from university-land so that was hard, and I think [my] not really understanding how corporate businesses work, their touchpoints, and what makes sense for them versus what makes sense for us as researchers and program designers…” {260, nonprofit, Asia‑Pacific}  “We’ve had a lot of technology companies coming to [our healthcare institution] and wanting to develop their products further and not starting from the essential needs that need to be met in [our clinical units] and then that kind of leads to a sort of non‑satisfactory outcome for all the people in the partnership…” {259, government, EU+}  “..all this sounded very exciting [to other countries’ health and development aid agencies and from their] point of view maybe it’s, ‘Okay, maybe can we get twenty million [to partly co‑fund] this particular project by tomorrow?’ But [here] this is not how things work! So need to temper that expectation. […] You have to take a longer-term vision and really understand how [this country’s] system works. Once you understand it and then build a trust, build a presence and show you bring value – then you have an opportunity to start influencing. So you cannot just come in and try to impose what you want to them. That needs to be understood and understood very well.” {203, nonprofit, Asia‑Pacific}  “There’s no pharma that I’m aware of that’s really doing a good job [with digital] so this is an opportunity. I think there’s some ways – certainly the startups would be very keen […] Right now they’re talking past each other. The role of a start-up is to figure out what their partner needs […] and it’s not easy to do so the onus is on the start-up to get their act together. But when the other side is not culturally attuned [to digital it is even harder and] it takes those outlier startups to have their antenna out.” {211, industry, EU+}  “And also they said, they came across that, ‘We have all this knowledge and we’re going to help you,’ and they showed me this tool and I was like, ‘Not going to work – not going to. You’re coming across as like from a tech lens solution and here’s how the [emergency ward] works.’ They went for the solution without truly understanding what the problem was. They solved the wrong problem and their solution wouldn’t work at all.” {247, nonprofit, North America} |
| Theme  Alignment not given more probing, careful attention upfront  e.g.,  - too much assumed, not enough methodically probed and clarified  - internal support within other partners too easily assumed  - too little time, effort spent to detect and consider or resolve issues within the initial discussions, negotiation, formal agreement; as a routine hazard or even tendency  - consequent misapprehension of alignment | Illustrative quotes  “[These partnerships] are the ones that I think most often just, beneath the marketing hype they just fall short […] I think the reason they fall short is two things. There wasn’t really an understanding of what the alignment was on the strategy between the two [partners]. They have a broad category where they say, ‘We’re going to work together, change the world on this topic and it’s going to be wonderful and it’s going to be very strategic,’ and that’s all very well and good, but that then requires a lot of substance behind it as to what are these projects that we’re going to work on […] and what exactly is our strategy as a [large research and healthcare institution] and how are we implementing that, and how does that fit with a pharma company strategy we just said we’re going to do a strategic partnership with? Are our two strategies really aligned? And are they going to stay aligned over the next 5 or 10 years? […] The ones [like this] that don’t [work], I think they’re generated too quickly. People just want a strategic partnership, it just sounds good…” {257, nonprofit, EU+}  “When you don’t do your homework, you don’t get commitment from all stakeholders involved in a partnership – no matter how good it looks on paper it all falls apart when the rubber hits the road. [We neglected] to understand the situation of the soft middle of the organization. […] It’s not just about going across the top C‑level individuals that have span of control over what needs to be done but you have to go a little top down and bottom up or at least middle up – understand both sentiment and commitment among the rank and file [middle managers] who will actually be driving things forward.” {242, industry, North America}  “One of the things with that collaboration – it was never really clear who owns the overall outcome from an [authorship] perspective, and I think everyone came to it with an intent of, ‘Well, that’ll just be obvious,’ but I think that has to be fairly explicit. And I actually have seen with other collaborations when there are tensions it’s this issue around scientific authorship. Although it’s very uncomfortable so people tend not to deal with it upfront – you have to discuss that really early in the process otherwise you end up with a lot of energy burned [later]…” {220, industry, EU+}  “..and now everyone tries to get all the buzzwords in, everyone is reading something different out of the buzzwords and we think we talk about the same thing but in reality we don’t […] and you’re not taking the time to completely understand where is your partner coming from—where they want to go and what they want to do—then suddenly you reach the point where you say, ‘But that’s not what we agreed,’ and the other one says, ‘Oh yes we have, look at the contract.” {245, nonprofit, EU+} |
| **Organizing theme**  **Alignment time-consuming to establish and uncertain** |  |
| Theme  Negotiations, alignment building, contracting involved seen as: difficult, prolonged, vulnerable to failure, too time and resource intensive  e.g.,  - assembling the partnership is anticipated/experienced as quite demanding, challenging  - may fail to align, reach formal agreement, or sustain it—and the perceived risk of this  - partly given the nature and degree of misalignment (see also theme ‘misaligned underlying interests, aims, incentives of those involved’)  - the negotiations, contracting involve too much time, expense  - transaction costs seen to undermine feasibility, value | Illustrative quotes  “So of course in that partnership negotiation you need to come together on alignment on many different objectives […] so how do you make that come together? That is a big alignment in the partnership negotiation. […] So it is very challenging because [these groups and their overall aims] they’re so different so coming together, that alignment building, making compromises, making trade‑offs […] that alignment building, expectation alignment is very hard.” {203, nonprofit, Asia‑Pacific}  “The ability to align all these things and the underlying motivation are tough. You have just really a lot of upfront work and […] if you can share the gains and if those could be perfectly distributed then there is a net gain, but you have to have alignment, you have to have trust, and, by the way, you might not get that alignment and trust.” {233, industry, North America}  “..once you start to go to legal and contract stuff then you’re losing agility, you’re losing some kind of productivity – this could be – if the legal parties don’t agree on a couple of things this could kill a project. And the managers might just say, ‘Okay, this is too much time internally to convince people who don’t want to do it.” {244, industry, EU+}  “..there’s always going to be issues around [intellectual property] ownership not because the academic actually cares but because their institution does. […] It wouldn’t be scalable [for us] to negotiate for nine to twelve months with every [institution] where somebody wanted to do one of these things, there’s legal costs and all that…” {230, industry, North America} |
| Theme  Cumbersome pace, processes of universities, major corporations, government bodies  e.g.,  - unwieldy processes, slow pace of institutions, of work with them  - their internal decision-making | Illustrative quotes  “Cumbersome internal processes on both sides, big pharma companies and universities.” {269, industry, EU+}  “..as an institution [the university in question is] incredibly hard to form a partnership with […] their processes are almost unconsciously set-up to prevent partnership…” {210, government, EU+}    “..with this [other government department partner involved] there is more bureaucracy and there is an extra administrative layer that actually slow down our work [with the other partners].” {238, government, Asia‑Pacific}  “..not succeeding in getting things through [their own internal] decision making procedures, actually making things more complex instead of simple […] and the complexity is not just in [large medical-technology corporations’] technology but also in [their] decision making also the contact persons, that starts to be really difficult and an energy drain. […] So the promise is there but they don’t deliver, with these big corporates…” {250, industry, EU+} |
| Theme  Turnover of senior leaders, partnership leads, liaisons at partners  e.g.,  - new senior leaders discontinue, reduce support for partnership, previous commitments  - expectations of turnover and consequent problems as factor in partnerships' perceived risk  - departures, flux of leaders, champions, liaisons involved disrupts partnerships’ continuity, work, relationships | Illustrative quotes  “Initially it went very well and then because of a change in leadership on their end and [the new senior] leader’s style that partnership has gone through some rough times. The motives changed because of a change in leadership […] he says he recognizes the value and he wants to continue the relationship, but then his actions are half-assed.” {202, nonprofit, Asia-Pacific}  “And getting buy-in from the [health] ministry on an ongoing basis – elections happen and the new [minister] comes in and sort of goes, ‘No, not needed’ – how do you deal with that?” {218, industry, Asia‑Pacific}  “And then [to continue describing negative factors] over the years quite a lot of change – personnel changes in these [partners] happened and these people were responsible for this partnership. If every half year one person out of these [few] is changing it’s difficult to let the [new] one grow into it, to follow the ideas, to incorporate the strategy of this coalition.” {245, nonprofit, EU+}  “One of the challenges for us in working with [such organizations] has been you tend to work with individuals [serving as senior leaders] rather than the [organization] per se and individuals move around with great regularity…” {256, nonprofit, Asia‑Pacific} |
| Theme  Too many partners involved  e.g.,  - the more partners in a partnership the more difficulty, complexity, time, negotiation it tends to involve, is anticipated  - hampers progress, prospects for partners’ alignment, engagement, success  - various numbers of partners were cited as difficult: more than two, three, four, five, or six partners, larger groups | Illustrative quotes  “Some of it’s down to just the practicalities of once you start involving more groups in a coalition inevitably the work gets more complicated, it takes more time to get agreement […] the dynamics just get more complicated the larger these coalitions are. […] The members of a coalition might just get frustrated with the slower pace and think perhaps it’s not worth their investment of time and energy.” {234, nonprofit, EU+}  “This is primarily managed by the [several] CEOs of the [several] organizations – so you start to have different levels of interest from the respective groups. That’s where having a partnership between multiple groups is very challenging. When you have a partnership between two organizations it’s quite easy because there’s an element of consistency and it’s easier. When you start to come up with a group and it’s [several] organizations […] it’s starting to be a bit more complex…” {252, nonprofit, EU+}  “…I think the number of people and the power of the voices that are in that room. It just seemed like we could have been moving so many more things […] We're all there representing another entity or set of priorities so I think sometimes that makes it hard to move things forward when there’s that many voices at a table…” {231, nonprofit, North America} |
| Theme  Resistance, limited cooperation from within partner’s organization and structure  e.g.,  - driven by conflicted interests of internal structure, siloes, and diverse agendas involved  - internally, by managers, staff, units, departments, and so on  - noted alongside limits to positions and authority of the partnership interlocutors, champions, liaisons concerned  - amid reactions in vein of 'not invented here', 'not one of us' | Illustrative quotes  “Well, within companies quite often they have conflicted interests either commercially or organizationally, actually there’s a lot of that which is simply the resistance of internal structure to partnership because quite often the partner is treated as a foreign body, in a very biological sense. And the organism kind of rallies around to protect itself. So that’s one [negative factor]…” {246, industry, EU+}  “..this [was] kind of a disconnect between the [partner’s] IT and clinical siloes. […] I think this happens pretty regularly – you bring in a whiz bang technology […] but then no commitment of resources or bandwidth to actually do the intervention. […] At the executive ranks there was a nodding of heads on the clinical side which turned out to be a false positive, when you went down to the VP and director level they didn’t have the bandwidth and it wasn’t a priority to them – and they had enough agency they could gum up the works and make it unsuccessful.” {242, industry, North America}  “..there’s a difference between power and authority, so [one of the most senior officials at the partner university] actually has authority but has no power [they] cannot tell people what to do. So I know [them] I meet with [the official] – that hasn’t unlocked what I thought it would. […] I can form partnerships with the most senior people in the university because my job enables me to do that, but it's not turning into anything because they can't translate it down...” {210, government, EU+}  "I think the main barrier was the [clinicians] in the other hospital weren't so willing to teach our residents. They prefer teaching their own residents so our residents went there and felt like they [were not welcome]. I think that was the main problem. It wasn't any financial aspect. They weren't their residents. I think that's it." {214, government, EU+} |
| Theme  Difficult for government bodies to collaborate with each other  e.g.,  - in general  - in contrast to how it is easier for companies to do so  - hard to reach agreement, in context of partnerships  - tendency to pursue their own narrower agendas, mandates | Illustrative quotes  “I have [over thirty local governments] in my patch – they have varying responsibilities, varying levels of interest in what they can do around health [topics] and trying to achieve collective approaches is really hard. Even with [smaller] groups of them it’s very hard, I guess because it’s very difficult to reach agreement organizationally. […] You need the ability to make representative decisions, and if you’re there on behalf of an organization you need to be able to – because you’re in the room […] you need the people in the room [representing each partner] to not be totally out on a limb – and that’s really difficult when the organization behind them is complex, decisions are difficult, there isn’t clarity or unity of purpose. Public sector bodies have more difficulty with that – so the chief executive of a company not absolutely but in large part can make decisions for that company. But public sector bodies we are communities of voice…” {209, government, EU+}  “I think the biggest problem we have to work together is the state agencies’ collaboration – which to me is really weird because we are all working on behalf of [the] people and it’s kind of weird different agencies could have different opinions. […] We are finding it very difficult to agree. […] The public sector I think is too siloed. The first point is to say, ‘Not my job, our agency is about this, it’s not about that. We are doing innovation, not research. We are doing company forming, we are not doing innovation’ – that kind of weird dogmatic thinking.” {265, government, EU+}  “[In this government body] we are really focused on healthcare but we all know health outcomes are linked to so many other drivers like education, environment, water quality, housing […] so it's very important for us to develop partnerships with the other government bodies. It might feel something easy but it is not because everyone has his own agenda and collaboration is really a challenge.” {228, government, EU+} |
| **Organizing theme**  **Challenges of efforts involving newer or less familiar areas and approaches** |  |
| Theme  Partners lack competencies, capabilities vital to their roles, the partnership,  e.g.,  - mismatch between partners’ experience, core competencies and nature of the partnership  - involves newer areas, approaches and/or ones less familiar to some or all partners  - underperformance, delays due to various partners’ limitations in terms of relevant competencies, capabilities, experience | Illustrative quotes  “..and it’s also not the bread and butter for any of the partnership agencies. The local [government] and [agencies] and businesses and [healthcare groups] are not really in the business of designing health promotion activities and [these public health initiatives] and evaluating the benefits for health. So even though we’ve tried to bring in some of that relevant expertise I think that’s made it harder as well.” {254, nonprofit, Asia‑Pacific}  “..some of the problems coming […] to our level the strategic level where we can see the companies too are unsure – they think that they work in innovation but they’re still stuck in old structures. They work in divisions or siloes, like cardiology or surgery, and they don’t have some kind of [innovation] team advanced to all the divisions. […] For example if you work in [a medical specialty area] and you have this really big need to solve something in healthcare, they will send all the experts they have in [that area] but they don’t really understand how to work with innovation. […] They lose time and we are losing time, money, patients – I think this is kind of the big takeaway and I haven’t really seen, and we have really big partnerships, and I have not yet seen, not even the biggest companies, who really can take another way…” {258, government, EU+}  “[They are] just some nice young people who are still on PowerPoint […] the really tiny start-ups, and you think they’re nice and sympathetic and contribute to a social better world and these are really nice people to work with, but they don’t deliver and you don’t succeed in getting some sort of business rationale in there and some sort of execution rationale in there...” {250, industry, EU+}  “..I think at the moment the big institutions and big companies are needed to provide that [necessary stamina] – on the other hand they’re not agile enough to provide the technical edge and the speed needed on the technical side to provide value.” {255, industry, EU+} |
| Theme  Risk-averse attitudes, people afraid to try something new    e.g.  - overcaution and hesitation to try things, use trial and error, accept some risk  - preference for familiar over new, unknown  - as common tendencies | Illustrative quotes  “I think when you’re doing something new, people can be so risk averse about it […] suddenly all these barriers emerge – and it’s actually, we do this all the time, we [already facilitate something close to this], all of those bad things might happen. So I think there’s a lot of hesitation and risk aversion to trying some of those new things…” {254, nonprofit, Asia‑Pacific}  "So I think in general the executives within most pharma companies are afraid to do things on their own that are outside the norms of the pharma community, and when several companies are doing similar things [scientifically] then everybody wants to get onboard and do the same thing […] It’s a safe route, but it’s not always the one that’s going to pay off the biggest in the end." {251, industry, Asia-Pacific}  “It was very simple in my view but it was not possible. People tried to approach it from a risk averse perspective. […] Everyone likes the status quo ideas, they don’t like change because it brings some uncertainty to you and how you manage this process together. […] How you can change is try small, not necessarily go into scale right away – and not to be afraid of it – very often [another thing] which prevents these partnerships, what prevents these ideas especially people in biomedical sciences they are always accustomed to evidence-based decisions, ‘Okay, if you persuade me with the evidence.’ That works in clinical medicine but if you are dealing with the macro issues […] it basically has to be by trial and error. That’s how the airplane was invented – there’s no evidence that this thing would fly unless you try to fly it.”  {219, industry, EU+}  “..and I think this is incredibly creative and innovative – and people are a little afraid of creation and innovation! People like to do things that they’re used to.” {260, nonprofit, Asia-Pacific} |
| Theme  Business models uncertain, yet to be determined, inadequate,  e.g.,  - ambiguity, unfamiliarity of new business models, business cases, related considerations and unknowns; estimation not straightforward  - not defined, to be negotiated  - unattractive from viewpoints of some partners, in terms of their expectations and perceptions | Illustrative quotes  “...one of the barriers would be just understanding, having a very clear understanding what’s going to be the outcome of your partnership. Because it’s like a new field it’s more like an innovative thing – so sometimes the barrier is, ‘Okay, [if] we do this together what’s the business model, what’s the impact, what’s the revenue?’ So that’s the first barrier.” {244, industry, EU+}  “..they’re so focused on return on investment, business canvas models, you want to have a business case, you want to know exactly – I would say, ‘We don’t know but you need to trust, if we are in this [innovation R&D] partnership with you we will do everything in our power to frame it because we need to create value for our patients as you need to create value for your company,’ and this discussion always takes – can take a long time…”  {258, government, EU+}  “..so I think scaling up and making it sustainable and coming up with a workable business model are challenges we need to face. […] The lower income people who really need it, they may not be able to pay for it […] so this is why I say the business model is important, whether it is reliant on philanthropy, whether the government can support in some areas, whether the insurance can support in some areas – how do we get it such that those who need the service do not find financials as a hurdle and those that provide the service don’t find financials as a hurdle either?” {223, government, Asia‑Pacific}  “What [business] model could be remunerative to the health system in order to incentivize them to prioritize the whole innovation arm of the business? It’s the classic free rider [problem] – ‘I help this start-up and then they sell it to all the other [US-based] health systems, what’s in it for me?’ […] So I see health systems as the best potential partner for the start-ups and we haven’t yet found the perfect melding to make each one benefit – the benefit’s very clear for the start-up, but for the health system [it’s more nebulous].” {211, industry, EU+} |
| Theme  Interested and suitable partners hard to identify, not aware of each other  e.g.,  - difficult to identify, approach, vet partners in newer, less familiar areas  - one or both groups simply not aware of the other | Illustrative quotes  “Some of it is just finding the organizations that do this kind of work. Certainly academia is such a big and disparate kind of an enterprise, every top medical center every top university is probably doing some research in this area. The question is how do you find the centers, how do you then find the investigators who are involved in it, then how do you actually go about working with that university to set up some ability to have dialogue and vet them so they understand the [technical] properties and qualities, and align on the contractual terms. That process can be really complicated.”  {225, industry, North America}  “One [barrier] is just knowing that [we are] there.” {230, industry, North America}  "We’re not always aware of the opportunities that are available. Not only [to partner with an] organization but also within an organization – so that’s one of the challenge we have […] Some of the [large] companies we have been working with I don’t think all the departments are even aware what we could provide them through the [existing] partnership, and so there’s an element around how your point of contact is integrated within the organization so then the organization fully benefits from it, so I’m thinking primarily of [large companies] where you have so many divisions, and you know that at the end of the day there may be one [person in the company] having very difficult questions to ask and doesn’t know that he could get the answer by going [to us] through his colleagues. […] I sometimes [also] find it strange when you have within the organizations people that are working on [topics mainly to do with our field] but never considered approaching us because they were not aware there was [a well-established association like us]…” {252, nonprofit, EU+} |
| Theme  To yield results only in longer term with notable uncertainties, upfront investments involved  e.g.,  - the partnerships’ projects entail relatively long timeframes, are perceived to  - key results, benefits expected only in longer term, are less tangible  - involves uncertainty in terms of unfamiliar, more speculative approaches  - requires expenditures and time in the present, on sustained basis | Illustrative quotes  “..the second [negative factor] is willingness on the pharma [company] side to invest in something like that where it may take a long time to get deliverables and is a long way outside the [particular scientific] paradigm that exists in the US or Europe…” {251, industry, Asia-Pacific}  “So there are a few things we are working on and they are proving to be such a challenge – it’s all based on this revolutionary product, that mentality of it. […] I think from [our internal corporate] perspective it’s one of those things like, ‘This is not the only thing I need to work on, I still need to make money [today] right?’ So it’s the prioritization – or actually more the appetite of how much time, not just money, time as well as human resources, in terms of what people are willing to spend knowing it’s going to be a long-term commitment to get to that goal – so that from [our internal] perspective is a huge challenge. Whether it’s our skillset, I think in terms of [such and such] it is still our skillset but the [other] piece of it […] and all of these things are common challenges that we face for a long-term project that has long-term impact…” {218, industry, Asia-Pacific}  “The growth in costs for healthcare has been unsustainable and a lot of pressure has been coming from the government on [healthcare organizations like us] to reduce their costs. […] If you reduce the amount [of healthcare provided people] will feel that pain quickly but if you just don’t progress a [long-term prevention] initiative like this it’s harder to see […] So I guess constant pressure [to reduce costs] and real difficulty in evaluating the benefits of this partnership, so the ability to say, ‘We did this program with [partners] to help these children [live more healthily]’, for that to translate to a reduction in [morbidities] in thirty years’ time, that’s a long bow to draw…” {254, nonprofit, Asia-Pacific}  “You have just really a lot of upfront work and investment. […] ‘You’re telling me I’m going to invest now and three years from now you’re going to give me that times twenty percent? Hmm, I don’t know, let me just stick with my current business model and at least I know what it is,’ that’s basically what people choose, right?” {233, industry, North America} |
| **Organizing theme**  **Limited overall resources and influence of partners, funding hard to come by** |  |
| Theme  Limited resources of partners’ own organizations,  e.g.  - pressured budgets, capacity, funding, staff levels, divisions  - small organizations, nonprofits  - larger organizations with limited budgets, flexibility | Illustrative quotes  “I think the key barrier for us as a not-for-profit organization is simply capacity. It’s not necessarily will. We have will, we have vision, and we have strategy. But capacity and resource capacity […] we’re pretty under resourced. Colleagues say to us, ‘Oh, you punch very much above your weight’ – and a lot of that is through the partnerships we have, you know, we have people doing work for us out of goodwill. But capacity is a substantial barrier to forging even stronger partnerships, for us.” {256, nonprofit, Asia‑Pacific}    “There are a lot of benefits to having these [disease-specific nonprofits] and being able to interact with them, to work together with them. Since there are very few of these organizations [here] and they’re not well organized and they’re not well funded, so we don’t have so much interaction with them.” {251, industry, Asia-Pacific}  “We have shortage of places, of beds, in all wards. There’s always not enough. And staffing is not enough always the budget is not enough…” {214, government, EU+} |
| Theme  Hard to come by funding for the partnership,  e.g.  - fundraising a pivotal challenge, competitive  - potential funders unconvinced, limited  - yet to secure funding  - available funding too limited, inflexible | Illustrative quotes  “Funding is very difficult part. We need very tenacious discussion with government people [to obtain funding for this partnership].”  {201, nonprofit, Asia-Pacific}  “Budgeting. I wish the [funding agency] would say, ‘Hey, this has big potential.” {215, government, EU+}  “[I am also involved with] the university and we have a very prominent donor who is interested […] but we’re not quite there.” {211, industry, EU+}  “Funding. [The local public health agency] has suffered a lot of funding blows in the last ten, fifteen years and some of the new monies they’ve gotten have been very targeted […] for very specific things. They don’t have as much latitude [as they used to].” {240, nonprofit, North America}  “..the coalition is constrained by funds […] They treated themselves as separate from the [regional government] and they were but the funds for the work they were doing were still coming from the [regional government] and I think that’s limiting. Everybody needs funding but sometimes it ties your hands – a lot of times actually...” {231, nonprofit, North America} |
| Theme  Partners not influential enough, too small, relatively unimportant  e.g.,  - actually or anticipated as such given the context, aims  - themselves, even collectively, in general  - relative to other partners, those considered  - represent groups, professions with less power, influence than others in health sector, society | Illustrative quotes  “..unfortunately on our own we’re too small to try to influence that agenda […] We are just one voice, we are [the constituency we represent], we’ve got an extensive membership, but we’re one voice among many because there’s all sort of [health] lobby groups…” {264, nonprofit, Asia-Pacific}  “..the university is a [huge and wealthy] institution […] and it runs very well, it’s run very entrepreneurially but […] I just bounce off […] I'm just too piddly, I'm too small, I'm too unimportant.” (i.e., the organization they lead is too small and so on, relative to the university) {210, government, EU+}  “..it’s always a battle to get that group [and related agencies] considered more in [our medical establishment’s] mainstream. […] I think it’s about a lack of understanding, a lack – people [from that group] don’t have the same power in [our] society that other groups do, so there’s plenty of very inspiring leaders but […] sometimes it’s a barrier to the halls of power.” {261, government, Asia‑Pacific} |
| **Organizing theme**  **Value unrealized or unclear to some concerned** |  |
| Theme  Value unrealized, intangible, not apparent to some concerned  e.g.,  - from perspectives of various partners, internal colleagues  - in terms of benefits to partners, advancing their aims  - value less than alternatives, hard to assess, not so tangible, undemonstrated  - in terms of perceived, actual, anticipated value | Illustrative quotes  “It’s [hard] having the mandate from management […] which means if the typical admin manager responsible for the [health services] budget trying to sort out all the figures and see if they balance in the end they sometimes look upon us and say, ‘You just cost money,’ they will never see the aggregated learning […] the effect of what we [did] by building capacity actually popped up in a totally different other setting than the admin manager would see […] we get the question a lot, ‘How do you measure your success?’ very many times I understand that question comes from the product-logic thinking – they want facts on how much shorter is the queue what is the revenue of this but right now [our] impact is something entirely different.” {227, government, EU+}  “..the [other company] didn’t think this was big enough for them and said, ‘Maybe we don’t need to do this we have other things to do and this is really low on our priority list we’re not making money out of this and we would like to,’ you know, the corporate [perspective]…” {218, industry, Asia‑Pacific}  "I see enormous value but it’s been difficult to move the needle [internally] and I think in large part it’s because [the partner would be an association in] nursing and it’s not medical, it’s the world over anyway, the culture is still very much around medicine and doctors rather than other sectors of the health system. From my perspective that’s going to have an impact on whether someone in [our] organization is going to go, ‘Well that sounds like a great proposition let’s put some money behind it.’ So there just hasn’t been that level of interest. So if I’m talking about [the medical associations] that’s a very different conversation […] You know the natural result is to go to the areas of greatest influence first…" {263, government, Asia‑Pacific}  “..I’m trying to convince [my institution] and we still have not, we’ve got to show them that it’s worthwhile for them […] It will happen once we have our first exit, once we have our big story then it’s going to come out all over and then they’ll make it work […] If you don’t bring the money then it’s all theories and thoughts and ideas, right? When you bring the cash then that’s when you’ve shown that it works […] I just have not been able to bring to exit [yet] to show this is not just a good idea." (n.b., ‘exit’ refers to a successful sale or partial sale of a start-up, when early investors/incubators can realize profits)  {Shashar 215, government, EU+} |
| Theme  General sphere of activity not a priority to some concerned, including partnerships related to it  e.g.,  - for organizations, internal units, individuals, broad group  - general sphere of activity (i.e. broader field, topic, area…) the partnership pertains to not seen as relatively important or worthwhile by some concerned, leading them to avoid or limit participation  - key parties do not consider mooted sphere of activity within their scope or priorities | Illustrative quotes  “And then basically what happened, which is like typical hospitals, is this person who was in charge of [their work in our field of activity] also got given seven other jobs. The first thing to squeeze out is [this kind of work] because there’s no line so while [this person’s] intentions were fine the conditions under which things proceeded didn’t match when we started and it’s not like [this person] didn’t want things to go, it was just the resources and the model wasn’t there.” {216, nonprofit, EU+}  “This is a huge market and in America [this part of the world] is barely a blip and they're not really seeing the potential […] so they’ll have people here and they’ll put money here and everything else but I think the people who are [here] feel like they’re still pulling teeth every time they're getting budget from [corporate] headquarters in the US.” {206, nonprofit, Asia‑Pacific}  “..[in this country] there is a mindset within academia that pure science is the objective of academia, not applied science. There is a sense of pride in doing pure science that doesn’t have an applicability […] That kind of mindset also prevents [researchers] from interacting with each other. For example if you have one interesting technology that might be combined with another interesting technology to make a new drug – for example a new inhibitor plus a new drug delivery device – there’s not really so much ambition or activity in trying to talk with other people and see how your ideas can complement each other.” {251, industry, Asia-Pacific}  “..if we did get to a point where we could do some joint campaigning that could be really powerful […] but it might not necessarily align with what our organizational priorities are, like it probably wouldn’t ever be a priority for our organization because [our members] they’re very much focused on the health policy kind of thing rather than what happens in [an area outside the health sector] or those things…” {234, nonprofit, EU+} |
| **Organizing theme**  **Relationships negative or insufficiently developed** |  |
| Theme  Relationships insufficiently developed, limited in part by the time and practical challenges involved to cultivate them  e.g.,  - (pre-existing) organizational, interpersonal relationships are too immature, insubstantial to support creation, functioning of partnerships noted  - time required to adequately develop, nurture relationships, and related practical issues like staff availability, travel, costs  - in situations where longer relationships helpful or crucial  - relationship and trust building limited by practical challenges to sustained engagement with key individuals, counterparts (see also theme ‘turnover of senior leaders, partnership leads, liaisons at partners’) | Illustrative quotes  “..and [it’s also due] to the quality of those relationships. Those relationships aren’t necessarily there and you have to invest time into developing them and that may or may not be easy…” {234, nonprofit, EU+}  “..people jump on [those partnerships] very quickly because it looks good, and there’s not enough of a relationship behind it, there’s nothing.” {257, nonprofit, EU+}  “I don’t necessarily have the time to explore new relationships or nurture the ones that we have […] the constraints are about the timing, having to constantly negotiate ‘When are you in town? We should grab a coffee,’ you almost need to allocate some time in the diary to actually nurture those relationships or make new ones […] that face-to-face is still so valuable, and not just one meeting.”  {266, industry, Asia-Pacific}  “You have to spend some time – years – developing the right relationship with the right people at the [agencies] before they’ll sort of think of you as a potential partner. I just think of a partnership we have right now with a [government agency in one country] and this only came about after years and years of meeting at different conferences that they were also at, and talking with staff in [the relevant part of the agency] in terms of smaller projects, so it takes time and investment in those relationships and so for us as a small nonprofit organization we just don’t have enough people to just go and do that, and there are resource constraints about how many international conferences you can go to and network and do those relationships, so those are the limiting factors…” {268, nonprofit, North America}  “I think there’s a few [barriers]. The relationships – usually […] on our side [the point of contact] would be me but on [their] side there’s like [several] heads so there isn’t really a point of contact for [the area we want to partner with them on]. So I think that’s hard, just from a relationship standpoint to build trust…” {216, nonprofit, EU+} |
| Theme  Mistrust, insufficient trust  e.g.  - among partners, individuals, groups—in terms of mistrust, suspicion, hesitation to trust, insufficient level of trust, skepticism others may take undue advantage; relatedly, skepticism of industry/firms as felt within some other sectors (see also theme ‘skepticism of industry partners’)  - gaps between what is said by some and their anticipated behavior, realities, what they actually do—from the perspectives of others involved | Illustrative quotes  “It’s not about processes that are limiting [these partnerships] I think the greater thing is the mindset and the willingness to trust each other. Underutilized partnerships are usually because of – if we dig deep it’s usually a trust issue which then manifests itself in process issues.” {223, government, Asia‑Pacific}  “..what stands in the way of the partnership is a little bit of mistrust.” (n.b., in regard to another partnership, the participant commented as follows…) “The other barrier could be somehow trust. Because it’s somewhat related to regulation – regulation kind of reflects trust levels. We have more restrictive, preventive regulations [of business practices in the pharma industry] because the trust is not always there. But there is also other component of the trust is basically understanding each other’s visions what drives you as a company as a partner and having that [currently elusive] dialogue that might enable these type of partnerships [with governments] through changing the trust…” {219, industry, EU+}  “One of the difficulties we often have is that partnerships don’t start off on a good foot, you’ll engage in a relationship with a partner and then you’ll realize actually their goal […] That’s fine if it’s acknowledged upfront but it can actually lead to a lot of – because if our vision for the end goal of the project is not the same, if theirs is an exit and ours is to get to end of Phase 2 then often there’s a mismatch there and there’s always this sense of having to ‘manage the partner’ and actually I find that as soon as you have to even say that phrase the trust isn’t there anymore […] There’s not a lot of transparency, you don’t know what [their] board want and they say, ‘Oh we’re fully committed to commercialize this,’ and you’re like, ‘You’ll never be able to commercialize this so of course you’re going to have an exit at some point,’ and then you’re always waiting.” {220, industry, EU+} |
| Sub theme  People involved do not get along, work well together,  e.g.  - friction between personalities, lack of good personal chemistry  - ego-related problems | Illustrative quotes  “We did not get on well with some of the people involved…” {270, government, EU+}  “They’re always fighting […] everything is personal – so communication and good personal relationship is crucial for this [hospital] to be a success and it’s not always.” {214, government, EU+}  “..there are issues that have to do with ego…” {213, industry, EU+}  “The [organizational] cultures are different […] I think to the extent that you and your counterpart are open to work with that it’s fine, but if the counterpart’s not open to working with opposites and they’re kind of arrogant on their own values then it’s really not worth it.” {202, nonprofit, Asia-Pacific} |
| Theme  Treated like a vendor rather than a partner,  e.g.,  - highlighted distinction of partner versus vendor, of partner relationships as typified by working together in contrast to one-working-for-other in more vendor-like role  - as opposed to preferred relationship of being perceived, treated as a partner  - more perfunctory engagement, demanding style from group who sees other as vendor | Illustrative quotes  “We started very clear on what we commit to but [they] wanted to add more things but not considering the additional resources that would be required, not only financial but the time involved. My perception was they consider us more a vendor, like we are providing something rather than we are someone working together on something like this. So I think it’s always a challenge especially when the partnership is coming from [a corporate partner’s] commercial lens or group and sometimes too closely connected to a specific objective [and deliverables]…” {252, nonprofit, EU+}  “I think at first it started out where it very much felt like a partnership and then going forward it might – like they might have thought we were more like a service provider in a way, ‘Why don’t you just do it? Why am I devoting my time to you?’ And we very much see things as partnerships.”  {206, nonprofit, Asia-Pacific}  “Why I think it [has struggled] is it wasn’t fully messaged out what they were and who they were and are they a partner or are they a vendor, or are they one of us? And we in healthcare respond very differently depending on where they are. So when I first met them I viewed them more as a vendor, so I talked to them and I gave them feedback but I think I would have been much more invested in helping them if I viewed them as one of us or a very strategic partner.” {247, nonprofit, North America} |
| **Organizing theme**  **Lack of time given other work demands** |  |
| Theme  Lack of time given other work demands  e.g.  - busy day-to-day  - partnerships as something that comes on top of core role, work  - desired efforts crowded out by more important or urgent work, necessities of job | Illustrative quotes  “It’s not out of a lack of goodwill or anything like that, the reality of day to day work is everybody’s so busy you can barely keep up with your own work and then to have these partnerships on top of it […] One [negative factor] is absolutely the busyness factor.” {268, nonprofit, North America}  “..it’s just like I get so busy on my day to day job […] it’s because of time more than anything.” {266, industry, Asia‑Pacific}  “So that’s the kind of dialogue we need to have [with these companies] and we’re kind of fighting to find the time for this.” {265, government, EU+}  “..we’re trying to do some of that stuff and get more involved but there’s a lot of things that get in the way of really digging into that kind of work – there’s nine thousand other things on my to-do list.” {231, nonprofit, North America} |
| **Organizing theme**  **Government and industry face hurdles to joint collaboration** |  |
| Theme  Hard for industry and government partners to engage, work together  e.g.,  - difficult to work together  - lack mechanisms for sectors to engage  - lack of understanding  - private seller and public buyer dynamics, sensitivities and rules  - skepticism of industry (see also theme ‘skepticism of industry partners’) | Illustrative quotes  “...so it's difficult for us to work with [industry]. But we all know we're partners – that the future of health depends on their capacity of developing new products and putting them on the market, and our ability [as the government] to give them [guidance on our future requirements] so the development meets our needs, but we're not good at talking with them […] probably there must be some kind of mediation body so we can meet both the private sector economic objectives and the public [healthcare] sector’s objectives – that’s something that [was on a previous minister’s agenda] – to try to reconcile us…” {228, government, EU+}  “It’s mostly a matter of lack of common understanding because, I mean [the pharma company and the government research agency are two] very different worlds. In a way it’s easy to establish partnerships between peer pharma [companies] because there is a lot of common understanding. It’s almost like saying as long as we agree on what are the key milestones and how are we going to work together, what is going to be step one, everything is fine. Academics sometimes pose a challenge although mostly academics and universities are used to work with pharma, so they know, for example, limitations around IP ownership, so mostly that’s okay. With other institutions such as governments, et cetera, it’s quite often just a lack of common understanding that stands in the way.” {246, industry, EU+}  “One of the big constraints of the health system in [our country is the preponderant] amount of funds injected from government – obviously there’s a huge amount of scrutiny. […] If I think about the environment things are hard to move quickly. Start‑ups and people in tech are generally interested [to work with the government-funded health system]. The ability to have some impact on patient safety or health outcomes is quite attractive. There is a willingness [on the government’s part] to work with industry in that regard but there are so many boundaries and barriers it’s difficult to make that work successfully. There's just too many roadblocks in the way.”  {263, government, Asia-Pacific}  “I come from an industry setting myself and was kind of thrown off at first coming to [this government health institution] and not understanding this whole procurement thing and what a big thing it really is. We’ve had a lot of partnerships where we started to work together – and then you realize later on like one or two years down the line that, ‘Oh, this isn’t working because we’re not going to be able to buy it, because we have to go out to the open market. And those are the [partnerships] where you can really feel the tension in the air because you’ve created something but you can’t use it.”  {259, government, EU+} |
| Theme  Skepticism of industry partners  e.g.,  - within public-sector health institutions, agencies  - suspicion of for-profit private sector and industry partners, of their financial motives and ostensibly more superficial commitment to other/shared objectives, as seen by others in unfavorable contrast to their own worthier motives and commitment; concern on pharma’s profitability, conduct  - within academia; however mentions were more qualified, such as how it had diminished | Illustrative quotes  “..we don't naturally create partnerships with industry partners because we have a suspicion of the commercial sector. So there’s a hypothesis and working theory that says, ‘We’re here to save people’s lives and make them healthy and that is a much worthier cause than making money’ […] and that’s not blanket true there are physicians working closely [with industry] but there is skepticism within the public sector, it’s a cultural suspicion.” {209, government, EU+}  “We’ve got a relationship with [the government agency]. I think it could be tighter. […] Because we’re a for-profit there’s a skepticism, ‘You’re just in it to make money, you’re not really aligned with us.’ There’s got to be a belief we’re committed to similar goals. [With them it] is still evolving a little bit…” {253, industry, North America}  “..Pharma is the devil for them. With [that large public agency] it’s quite difficult – even if [their top executive] says, ‘Pharma industry is not a concern it’s a solution,’ but the reality is very different because some [people there] say, ‘No, don’t [collaborate with them].”  {221, industry, EU+} |

**Note on rights and permissions** The original authors of this document (“Additional file 2: Negative factors and illustrative quotes”) are Greg Zwisler, Christopher Sauer, and David Shoultz. The original source, for citation purposes, is their manuscript entitled “Vital lessons from struggling partnerships and potential partnerships: an international study with leaders across the health sector”, published by BMC Health Services Research. This Additional file and its contents are licensed under a Creative Commons Attribution 4.0 International License, which permits use, sharing, adaptation, distribution and reproduction in any medium or format, as long as you give appropriate credit to the original author(s) and the source, provide a link to the Creative Commons licence, and indicate if changes were made. To view a copy of this licence, visit <http://creativecommons.org/licenses/by/4.0/>.
