# Supplementary material for: Vital lessons from struggling partnerships and potential partnerships: an international study with leaders across the health sector
Source: BMC Health Serv Res. 2024 Nov 26;24:1470. doi: 10.1186/s12913-024-11944-7 (PMC11590265; doi:10.1186/s12913-024-11944-7)
Supplement: Supplementary file 3 — Supplementary Material 3. [file 12913_2024_11944_MOESM3_ESM.docx]

**Additional file 3: Success factors and illustrative quotes**

Listed in the same order as in the manuscript (Results, Table 3). Illustrative quotes are anonymized and disguised.

| **Organizing theme**  **Meaningful value for and from partners, responsive to key needs** |  |
| --- | --- |
| Theme  Win-win, partners respectively see benefits they value  e.g.,  - mutual benefit and interests  - benefits for individual partners, their own missions and aims  - as partners achieve benefits and successes this fosters continued participation, more joint efforts  - benefits important to partners | Illustrative quotes  “Fortunately for me a lot of them are politicians and they want to score points, take care of the poor. So we have alignment of interest. I want to take care of the poor, you want to score points, take care of the poor – you help me, you give [my initiative] visibility, you give [it] a profile…”  {243, nonprofit, Asia-Pacific}  “..so there’s a business relationship there that’s win-win […] If our core business didn’t add value to them and we didn’t feel it was a good [business relationship for us] then all that other stuff doesn’t happen. So at the core, the core business has to be a good business proposition for people, a good value proposition. […] We’ve prioritized it also and we’ve set up a cadence around these [additional] things [we’re doing with them] so we’re investing in the relationship as well. It’s beyond just a contract.”  {233, industry, North America}    “..and then the fact that [it's delivering results] it's working […] I'll just give you a concrete example […] That’s a great win-win because I need them to deliver my value proposition, they need me to deliver their value proposition […] Their innovation pipeline’s accelerated and high quality and has a constant deal flow, and we’re delivering on our mission to our startups. I think the fact it’s really clear what the complementary value exchange is, the stronger that is the stronger I think the partnership will be.” {216, nonprofit, EU+}  “So we said OK, we develop the compound now we reach the end. It will change completely [treatment of] the disease. This kind of partnership and it’s a very successful one – because it’s a win-win one – very important for [them] for their reputation they receive money but very important for [us]. It’s very creative. […] Because it was very successful and create some personal relationships, the [CEO of the partner] is a very good friend and [we] signed a new agreement to develop [another] compound…” {221, industry, EU+} |
| Theme  Partners respectively bring value, complement each other    e.g.,  - in terms of expertise, capability, credibility, direct knowledge, etc  - in terms of useful, vital aspects or strengths the others lack  - in part by matching on similar aspects, as when useful to have similar geographic footprints, etc  - evident partners can contribute, perform their contemplated roles | Illustrative quotes  “One is [our organization] really has the ability to add value on this – for any partnership you really want to bring complementary capabilities, in this case [our organization] clearly brings a lot of the technical value to the table…” {203, nonprofit, Asia-Pacific}  “..I think each party brings somewhat different expertise which have added value all the way through the collaboration. So [this partner] they bring for us two things – one is very grounded in the patient which is incredibly important, second piece is because they have been agnostic with who they collaborate with […] they have a sense for the field. And the [other partner…] what they’ve brought is the sense of how it’s different in rare diseases […] what sort of permissiveness can you get, what flexibility is there, what’s the room for innovation…” {220, industry, EU+}  “..one of the things that has been particularly productive about this partnership is that they, like us, have a really wide geographic network – and they have access to, so [the partner] is a constituency of constituencies so their member organizations are national [associations of their profession] globally so their reach is quite broad and shares the breadth that ours does […] The number of people that they’re able to access through their constituencies and the range of expertise […] makes them a huge asset in being able to share information back and forth […] and I think another advantage they bring to the game is [our] constituencies are [government agencies] and their constituency is people who actually interface with patients and bring that on the ground experience and on the ground validity.” {262, government, EU+}  “I guess this comes a little bit to some of the success factors behind it […] So in [this country] there has also been a [change] which meant that [everything involved on that end] was [consolidated under the aegis of our partner] agency – which is something other countries are quite jealous of. […] I think [also] probably my relationships with the people who could implement the results of this work were helpful as well – I’m [an affiliate] with [the professional association] and I’m [active] with [an influential membership organization], I’m [affiliated] with [another association in the sector]. All of those relationships with the people who could really use this information to make a difference helps as well – that you know the work that comes out of this partnership is not going to be filed away on a shelf somewhere, that it will be used and implemented. It certainly did help with achieving funding [from the funder] and the support of the [partner agency] initially that I did have a track record of being able to translate research findings into practice.” {254, nonprofit, Asia-Pacific} |
| Theme  Identify and respond to the right needs, proactively  e.g.,  - astute grasp of needs, choice of needs to focus on, in the context  - as opportunities to build valued initiatives, relationships, support  - of partners and stakeholders, with consideration to alignment  - to orient, advance the work of the partnership  - taking the initiative to do so, from early on | Illustrative quotes  “..getting the right needs at the right time, understanding opportunities, using visual cues, turning it into something tangible […] a concept that was the right balance, the zeitgeist if you like, of care is what we need at the moment.” {210, government, EU+}  “..they’re quite hamstrung around advocating for themselves. We’ve been able to understand the needs of these organizations for us and we’ve been able to respond to it in an agile way so the partnership is valuable to them.” {256, nonprofit, Asia‑Pacific}  “Having the stakeholder mapping really early, having alignment, having addressed the right needs…” {258, government, EU+}  “I directed all my energy, because I had a small team, to figure out what [our counterparts] needed [at the other partners]. To say, ‘Look, what are you struggling with right now?’ And they would say, ‘Well, my surgeon has no idea what he’s doing, I’ve got this clinical champion who is the wrong guy,’ so I could say, ‘Okay, [and help them]…” {232, government, North America} |
| Theme  Addresses pressing needs and challenges motivating partners, felt by them  e.g.,  - in cases of obvious necessity, acute frustrations, or more subtle yet still serious needs, challenges felt by partners involved | Illustrative quotes  “The policy had thrown a lot of money at problems because it had a lot of money [before our fiscal crisis] instead of really getting at foundational root cause issues which also plagued physicians in their practice but weren’t necessarily addressed and I think there was a sense money would fix all that ails their issues, which compounded the system issues […] So I was saying, the partnership aspect came from having a burning reason to come to the table where there’s skin in the game for each party...”  {236, government, North America}  “The [regional governments…] were so frustrated with the situation – they really wanted another solution of this problem.” {239, government, EU+}  “The head of psychiatry [said] they could not recruit people – they’re dealing with diseases that have a stigma so patients didn’t want to come to the hospital and be seen. So there was a clear match of a need, an unspoken need at the hospital with technology. So it really wasn’t technology looking for a user [...] [It matched] a bunch of different needs [the psychiatrists] had and that they had written down but never thought of using technology to help with that.” {255, industry, EU+} |
| Theme  Collaboration facilitated by a partner’s well-adapted size, structure  e.g.,  - enhances the relative ease of the partnership, its development  - partner not too big and cumbersome, too small and unestablished  - partner’s organizational structuring aid their functioning, suitability in connection with the partnership | Illustrative quotes  “By [that industry’s] standards it’s a small family-run company […] Small means a thousand employees. […] It to me is actually way more fun to deal with them the company than it is to deal with a well-known corporate company. […] They have so much less bureaucracy, they have less complication, their legal process is incredibly simple. […] I find it quite fun working with these small companies, and I think they have a lot to offer in the early stages of partnership and research and innovation. They are more flexible and they’re usually more open to the innovative ideas...”  {257, nonprofit, EU+}  “…it’s much more easy to build partnership with a structure like [our partner] than a big organization because the structure of [that large agency] is very difficult one.” {221, industry, EU+}  “Do they work in siloes or do they work with some kind of innovation management system? Because otherwise you will get sales and marketing first, that’s the first persons you meet, and then they may send some R&D people which can be good but may be even worse. […] [In this successful case our partner was] a big company with all kinds of different divisions, and after the first year they transformed their organization so they had one head of innovation across all the divisions, who had their own team, and that’s where the success came from. That was the success factor behind all of those activities.” {259, government, EU+}  “…if you have been treating [this disease] for a very long time at the hospital you have been caught with recurrences and so […] I think [the clinical partners] needed to see there was a serious company built around these creative [entrepreneurs-cum-patients] who had the idea. They needed to see that they got funding from others, from investors and the [support system] had them in a business development program, so it wasn’t connected to just them.” {255, industry, EU+} |
| **Organizing theme**  **Shared purpose and commitment, around humanitarian aims** |  |
| Theme  Shared purpose, resolve  e.g.,  - of partners, individuals involved  - common (larger) ambition, cause, or aims of those involved, advanced by their partnership  - collective effort, determination  - similarities in partners’ values, their respective visions, missions | Illustrative quotes  “..we were all striving for the same thing, we knew surgical outcomes could be improved, we knew there was an opportunity for change, but we also knew it was incredibly hard. […] I think as far as a successful partnership it was because it had a shared purpose. We all felt strong about the things that brought us together even though we were all from different places.” {232, government, North America}  “One is that it unites all the partners […] around a common cause, it’s not something that affects directly their revenues it’s not something they’re selling to each other but this is something they’re passionate about. […] I don’t always consider partnership what is called partnership unless this ingredient is in place and it is a core for sustainability, you have to be working toward the common outcomes rather than the transactions that [are just] the nature of their business. […] If you have a problem you don’t necessarily switch the partner, you try to improve each other and […] no one would say, ‘Okay, I don’t want anymore to be with [partner A] and [partner B],’ and try to pull out. We just help each other…” {219, industry, EU+}  “I went to this breakfast that they ran and their [chief executive] was speaking quite genuinely I felt about how we need health systems that are patient centered and people centered, which is obviously our reason for being. […] It really came about, and again this is where these sort of partnerships seed on reflection, is personalities and people who have a sense of common purpose.”  {264, nonprofit, Asia‑Pacific}  “..it was the people from the [regional governments] that had to negotiate with each other and come back to their friends their colleagues and say, ‘Okay, we have to change but we want to change because we want to go do it together.’ […] So it was a common idea of doing this together. And it wasn’t forced by our organization or the [national] government – it was the stakeholders themselves, the [regional governments] that decided this. And that was key…” {239, government, EU+}  “It shows that two different organizations – if they share the same vision to provide sickness care and to do it in the most cost effective manner, can do [this model] together.” {223, government, Asia-Pacific} |
| Theme  Personal involvement in humanitarian shared purpose  e.g.,  - efforts to improve access, equity, patient outcomes, other areas to do with human welfare  - felt across teams, key people or everyone involved in partnership  - inspires extra effort, resolve, commitment, perseverance  - attributed to peoples’ values and interests, compelling nature of the humanitarian purpose, familiarity with problem, patients | Illustrative quotes  “It’s a good mission. It’s giving the people who live in the periphery of [the country] better medicine. So everyone wanted it to be a success. […] Everyone was personally involved in it. Really it was like a mission for us.” {214, government, EU+}  “..there was a commitment to the patient which has permeated the entire joint team – so there was a reluctance to just give up because it was felt we really now had a strong commitment to the patients. […] What we did for this program was to actually bring patients into the teams right from the beginning and […] actually for the team it’s been a major driver – every single member of the team from the biology postdoc to the chemist to the toxicologist that’s what they talk about, that’s what resonates, they have actually seen some patients.” {220, industry, EU+}  “I think it’s probably down to shared values like valuing the same principles of access and equity and fairness. He would never use these words but it’s social justice.” {267, nonprofit, Asia-Pacific}  “..all the people I’m talking with who are [also] involved on a day to day basis either designing or because they’re a service provider to mental health patients, I think their interest is that they feel passionate about it actually – I think there’s more passion around mental health. […] Every time I do a conference and everybody puts a hand up when you ask them, ‘Has dementia touched you or your family?’ Dementia somehow tugs people’s heartstrings – so I think there’s an enthusiasm and a willingness to go the extra mile...” {235, nonprofit, EU+} |
| **Organizing theme**  **Professional relationships and trust, their cultivation and good basis** |  |
| Theme  Professional relationships, history with each other and accrued trust  e.g.,  - familiarity, networks  - longevity of relationships  - history working together  - relationships mature, trustful  - prior relationship building | Illustrative quotes  “I think it’s relationships. We’ve been lucky in our sector in [this country] people tend to know each other […] and those relationships tend to be there – and we’ve also been lucky in there hasn’t been a high degree of turnover in staffing key roles – so people who are chief executives level and director level as well. These are all people who knew each other for at least a couple of years and had worked together on various different projects before we actually made the decision to come together and formally form that group. And I think it’s about if you’ve got that relationship there and you trust each other and trust in sharing information...” {234, nonprofit, EU+}  “I think the other factor here is the longevity of the relationship, that these sorts of things just don’t happen overnight. It takes several years of talking about things and getting to know one another and building up the trustful relationship before these things do work out.”  {268, nonprofit, North America}  “And I think a lot of that boils down to – so I think we’re very proud of that, of those relationships. It’s taken a long time. We’re here in our sixth or seventh year and people forget the first eight or nine months were very much about suspicion and what are you doing? […] Takes time to build relations and hearts and minds and all that – but it’s been worth it…”  {207, government, EU+}  “..and all this usually based on the trust from previous activities so people know one another would deliver and be committed toward that.”  {252, nonprofit, EU+} |
| Theme  Actively build trust, relationships  e.g.,  - concious efforts to develop personal relationships and trust, especially from early on;  - by fostering personal familiarity, credibility, information exchange;  - by helping others, doing what you said you were going to do | Illustrative quotes  “I suppose I was very conscious of actually developing very genuine relationships where I genuinely sought their opinion and input into anything I was doing […] They really found value in that information exchange and sharing of ideas and I found I was able to get things implemented quite quickly because there was that real trust […] It takes all that extra time at the start but once you’ve established that trust and relationship it means that it’s much more productive and efficient going forward…”  {266, industry, Asia-Pacific}  “Connections, networking, and to build the trust. […] A lot of people invested in the story early on. I think to allow some time for design meetings and interviews, that’s not a huge investment, but it gave the time to build trust in technology and trust in the people. {255, industry, EU+}  “I think I developed a sense of trust. I’m quite authentic, and I would do favors, this is my mantra, I do things for people. I do things for everyone. I have no criteria, none. And I have learned, and the literature supports this, that once people know you’re in it for the right reasons and you’re willing to help, then they are more likely to return a favor and to have that relationship of give and take […] and they were willing to disclose really major [failures] without knowing that I would never reveal them to the powers that be. […] So I would always try to come at it from another angle for them that they didn’t have the power to do within their own organization [...] and I would speak highly of them, and that’s another favor quote unquote, because speaking highly of someone their nature their style, that spreads.”  {232, government, North America}  “…you have to do what you said you were going to do, you have to be trustworthy build a track record – beside all the nice meetings and catching up are we going to do what we say we’re going to do? Are we responsive? […] You build credibility and trust that way – and we have so there we go.”  {233, industry, North America} |
| Theme  Collaboration as peers and equals, of professional peers  e.g.,  - partnerships of professional peers, working together directly  - in the sense of working on something together as peers, with regard for others as relative equals, in partnerships’ context  - with power shared, partners’ input invited and taken onboard | Illustrative quotes  “I think one of the reasons it’s working so well is because this is done on the actual stakeholder level, it wasn’t done on the highest administrative level of the [institutions] where if we might have brought the CEOs of these [institutions] together and say, ‘Let’s do this formally and sign a contract for collaboration,’ this might have not worked. But this is done through the actual people that are leading [clinical research and practice in this field at each of] these institutions…” {222, government, EU+}  “..and a feeling of being peers instead of vendor and client.”  {269, industry, EU+}  “Number two, to be very ‘atypical [of a stereotype]’, which is [to say we chose to be] non-patronizing, non-dominating, non-aggressive […] it meant bringing in leaders of other organizations equally. And making it very definitive that is the purpose and very equal to others. […] Thirdly, it was a diverse partnership [of] professionals talking to professionals, so we found people who had been trained in public health had an easy way to communicate even with most conflicting organizations because of the common language of public health, same with nurses, so the [common] professional backgrounds of the key presenters helped a lot in building the partnership.” {213, industry, EU+}  “[Our counterparts there] really valued community voice and community-driven change so I would say the partners they were funding had an equal voice at that table in order to plan and implement the work. […] They had such a keen ear to community voice and community-led decision making that I think that’s what made that partnership successful.” {231, nonprofit, North America} |
| **Organizing theme**  **Strategic vision, support, and approach** |  |
| Theme  Strategic vision and approach  e.g.,  - compelling overall plans and guiding aims, goals, principles  - well-considered, accounts for interests and dynamics involved  - significant aims and opportunities, long-term  - pursued systematically, with discipline, through actions consistent with the strategic vision and aims, over time | Illustrative quotes  “[We determined that to develop these partnerships] required significant change to our staffing, to our board membership, it actually required a change to our charter as well so we had to think fairly strategically – and this was all happening in a fairly dynamic political space. […] So I think the essential thing was actually to take a strategic approach to this. Firstly to understand the change in political dynamics, identifying the opportunity required a very strategic vision from myself and my staff, and from the board as well…”  {256, nonprofit, Asia‑Pacific}  “It really was one of vision. Of understanding that what got us here isn’t going to get us to what we want to know. So it was having a view for the types of questions that people wanted to answer and the scope of ambition and getting people inspired to be a part of that frankly. […] Part of what made it work it wasn’t all centered around [the first big] goal, it was looking a couple of steps down the road, what’s next after that and what’s next after that – that made it necessary or compelling for people to want to maintain that level of collective work once that goal had been achieved. And in science they have to start planning for those ahead of time…” {230, industry, North America}  “…from the very beginning [years ago it was] meant to eventually lead to something big – not restricted only to a pilot – so it’s planned towards that, work [in our] way of always starting small, and then patiently rolling that, rolling that out [and now a lot of its elements are in the government’s national plan] – so having that leadership and that vision in mind from the very beginning rather than do things opportunistic here and there – I think that makes a lot of difference.” {203, nonprofit, Asia‑Pacific} |
| Theme  Senior leaders provide their support, clear mandate  e.g.,  - effective, decisive interventions  - executive champions | Illustrative quotes  “What made it successful was that at the very beginning two years ago we got commitment from senior leadership that this was something that we wanted to pursue […] We had CEOs from both organizations, senior VPs representing areas of each organization and that commitment gave both sides authority and mandate to go forward […] Within my organization I can kind of refer back, ‘Well you remember, the big guy said this.’ I think that helps all along the process, if we hit certain barriers, or if we needed legal or compliance to consult on something, they’ve got a million priorities so to say, ‘Well, we had this big summit…” {253, industry, North America}  “The person who was leading their work [with us in this area] was their chief [clinical] officer […] She happened to be somebody at a senior enough level that she could influence the organization towards [expanding] the work.” {268, nonprofit, North America}  “..the current president of that [partner] organization – who is really enthusiastic, a huge advocate, and someone who is really keen to just get on with it.” {263, government, Asia-Pacific} |
| Theme  Key financial commitments signal credibility, provide key resources | Illustrative quotes  “So that sparked it off—getting a national grant. [We would have gone ahead without it] but the mandate of having a national agency saying okay this is important — you’re doing cool stuff — that was really important.”  {227, government, EU+}  “..there has to be some level of commitment — since we’re an international company, from the global headquarters — that we will try to look for new opportunities in Asia and if we find something that looks interesting we will decide to fund it with enough funding and help as much as we can to get it into the global [research and development] portfolio.”  {251, industry, Asia-Pacific}  “So in [our country] we have things called partnership grants—which is funded by [a government agency] — and the aim of those grants is to create partnerships between decision makers, policymakers, clinicians and researchers. So one of the things that obviously helped this work was being able to see to the partnership grant — which is really specifically designed to foster and support partnerships like this.” {254, nonprofit, Asia-Pacific} |
| Theme  Effectively develop vital supporting partnerships, support of key stakeholders, internally and externally  e.g.,  - productively lining up support of internal/external stakeholders through concerted efforts  - in well-conceived, skillful ways  - with attention to the power and political dynamics involved | Illustrative quotes  “..for something that is seemingly around two organizations when you start to deconstruct the complexity of the partnerships that are needed across the table at individual and organizational tiers – actually that you also need internal partnerships within organizations to make this happen and the scenario I was in was that in order to ensure there was success at the table there were also partnerships one had to forge both informally and formally with the other geopolitical parties that weren’t necessarily at the table but the impact of decisions made at the table would impact them. […] I pulled in all my resources frankly to [accomplish this]…”  {236, government, North America}  “We can’t underestimate the efforts that have been necessary for the [corporation’s regional] leadership to get buy-in from the [global] leadership for the investment [to support their participation].” {265, government, EU+}  “so I managed to get to the elected lead of the local authority […] and he said, ‘Yeah, that’s a fantastic idea,’ so he and I persuaded the local authority to go back out to their main council. […] Meanwhile I went to my board [and made the case] they agreed so that was my partnership with my own colleagues with my own board (n.b., the local health system). We then started managing to get external investment – you know the Marshall Ganz stuff, ‘power over, power with’? Marshall Ganz helped Obama get elected and he did a lot of power mapping. Partnership it’s a very complex managed process. My [other] organization it’s called [‘Networks-X’] networking it’s a real skill if you get it right. […] We had identified – we were working with [the funding body] so we’d made a presentation to [their] board so we’d gone around scattering seeds. […] Big ambitions can be leveraged through energy, pace, and vision – but it’s actually a lot of hard work of sitting with white boards and mapping who has power over you, who do you have power with, what’s most likely to work for them...” {210, government, EU+} |
| **Organizing theme**  **The right people, individuals make the difference** |  |
| Theme  The right people, particular individuals’ involvement as pivotal    e.g.,  - emphasized by contrast to how if others had been in their roles success may not have followed  - credited for compatible personalities, their leadership, experience (c.f. next sub-themes)  - reliance, in part, of partnerships on the people involved, and good fortune as to who they are | Illustrative quotes  “I think if these individuals who worked on this project were replaced with different staff that I know and worked with at the [partner] it could have gone vastly different. So I share it to say it was this amazing partnership but the credit I don’t think is all due to the [partner] public health system so much as the folks who got to work on this project…” {231, nonprofit, North America}  “..so [they are] a very highly respected expert in the field, great relationships, great personality — so he has a lot of respect and a lot of trust. Very important to have the right people working on the partnership.” {203, nonprofit, Asia-Pacific}  “The [leader] of the [national] authority – who said, ‘I am willing to try a new model. I will do everything sort of innovative.’ [They are] a lawyer which means [they] know about the rules – [lawyers] can be quite inflexible because, ‘It is allowed [or] not allowed’ – but [they were] very flexible, ‘Okay, this is the edge of what we can do but we can do it.’ […] The government was sort of scared – so [their] statement that, ‘This is okay, I can try this,’ was important.” {239, government, EU+}  “He’s quite a champion and not necessarily of our [work] but just of [the technology] more broadly […] If it had been a different person who was the [partner’s] president […] it may not have resulted in such a good outcome and we’ve seen that with other [bodies] that we’ve been involved in. If the person isn’t much of a fan or much of an advocate then the story goes nowhere.” {263, government, Asia-Pacific} |
| Theme  Leadership by key individuals  e.g.,  - who are visionaries, inspiring, courageous  - provide direction, orchestration | Illustrative quotes  “I feel sometimes you just have to have the right people in the right place at the right time and that was the case here. [The executive leading this work at our partner] she’s just an amazing woman, a visionary, very smart with a very good heart and saw how this could change the way care was delivered and thought if we could do it [in their largest hospital] we could do it in other hospitals and [they] have a network of affiliates. She happened to be somebody at a senior enough level that she could influence the organization towards the work.” {268, nonprofit, North America}  “I think a lot of it had to do with the leadership of the project, so the people who were selected to be the co-leads, one from [our company] and one from [the partner university], these are people who are just fully committed to this project…” {246, industry, EU+}  “I think – [the leader] – [he] was the professor heading up our research team and I think his courage really, he was able to envision a project of this scope and scale, and certainly for me it was wonderful to have a mentor who rather than saying let’s do a study with 2,000 [individuals’ records] would say let’s do a study with [tens of thousands of individuals’ records] – and I think his experience, he was […] used to doing really large scale studies…”  {254, nonprofit, Asia-Pacific}  “From the [partner] NGOs one person was leading it, had a clear strategy in mind, others were following because she was convincing and there was enthusiasm about this. And because there was enthusiasm and they had success it was motivating for the others. They trusted her. So for the other ones it’s an easy game just to follow and add a bit more – if you have a good leader.” {245, nonprofit, EU+} |
| Theme  Personal affinity, working together well  e.g.,  - chemistry, friendships develop  - working together well related to affinity, respect, compatibility  - working together is enjoyable | Illustrative quotes  “It’s a group of people that when I come back to [the city] to visit in July I’m going to happy hour with them, and I say this to say that I think part of the reason it worked [was] because everyone at that table respected and liked each other on both a professional and personal level. So I think that made it effective and I think it’s also what made people really excited to work on it.” {231, nonprofit, North America}  “I think it comes down to how well you work with the partner […] it still comes down to the relationship you have. Just like in a start-up funding process – a lot of people do their business plan, well, if you have in front of you a partner you generally feel him or you don’t feel him. [And in the other successful] example I gave you […] again it was person to person. Not least because you have different kinds of person – it’s not rocket science but I think it has to be [noted]. Sometimes you could force it because the interest is so big you have to, but a lot of times if it doesn’t work out you’ll find another partnership.” {244, industry, EU+}  “..the people, the individuals we work with definitely make it the success that it is. They’re people we enjoy meeting with!” {206, nonprofit, Asia-Pacific} |
| Theme  Key individuals’ experience in professional spheres the partnerships inhabit  e.g.,  - the specific fields, domains, organizations, overall milieux  - their standing, know-how, grasp of the dynamics, relationships, familiarity with the organizations, ability to collaborate in the space | Illustrative quotes  “And the third was probably my part then because I had a background nationally and on the [wider regional] level as an initiator of design-driven projects [like this] – I’d been running [such] collaborations between industry and academia around user-driven innovation. […] Since I’d been working nationally I also had the opportunity and knowledge to apply for national grants […] I had relations with [the national funding agency] from before. […] Other partners we had onboard from start on – and that was [also] my part into it – [were the country’s] biggest design companies…”  {227, government, EU+}  “Partnerships often in my experience rely heavily on the people, not only the organizations and mandates engaged with them, and I think we’ve been quite fortunate of [our partner], who when I started here had a leadership who had worked [here] previously, and they had a specific role called [liaison] that was staffed by a person who had also worked [here previously], so they understood [our complex organization] particularly well and were people who understood the constraints we were operating in…” {262, government, EU+}  “[First of all it was] the people – so it was individuals who were highly professional, well-regarded in their own communities. […] Also, at least three had at least some international experience, so they knew what the UN is all about – on the technical side not the political side. They understood donors, civil society relationships, and they also understood the threats towards engagement with others. So [people who had] some genuine understanding of how to connect with other people, about cooperations.”  {213, industry, EU+} |
| **Organizing theme**  **Open communication, partners upfront and understand other partners’ perspectives** |  |
| Theme  Partners upfront about their respective objectives, expectations, concerns  e.g.,  - from the beginning, early on  - with respect to potentially sensitive topics, concerns such as partners’ interests, challenges  - core requirements, conditions | Illustrative quotes  “..from the early stage we had an open discussion with [the government partner] around what their expectations are, we’ve been able to be very open around what we would like to do. […] Another successful example of partnership we’ve had was with a pharma company where we and they [did certain things together]. The pharma company has been very open on their objective, we have been able to define clearly what we expect. […] In both cases there was an element of open discussions around what are the objectives, from the beginning…” {252, nonprofit, EU+}  “Some of that’s actually being clear on what expectations of all parties are and when I talk about expectations I mean what they can expect from me as well as what I expect from them […] so being really upfront about that.” (n.b., participant continued and, later, in regard to another successful partnership, said the following) “Also being like, that sort of awkwardness between, well, I think at one point it was kinda like, ‘Well, we’re not paying you so what’s in it for you?’ And then I realized, well, academics actually they need to be publishing research and running projects and getting grant funding so there actually was a lot in it for her to partner with us as well. So just being really open about that and having those discussions I think was really important early on.” {266, industry, Asia‑Pacific}  “If you don’t fix from the beginning the real objectives you will fail. It's why I can tell you from the beginning of my partnership with [them] it was very clear – they [laid out what they wanted to do and their three fundamental conditions] – ‘Do you accept or not?’ Was not easy – I remember when I signed the agreement during a press conference it was like, ‘Wow [the company accepted those conditions?]’ – ‘We’ll see [if it works]’. And we’ve seen. We [achieved real success and] we were the first one with this…”  {221, industry, EU+} |
| Theme  Active communication, information exchange  e.g.,  - sharing information  - accessible, responsive, reliable  - open communication channels, supportive systems and practices | Illustrative quotes  “I think the main thing is the communication. Communication is really important so there has to be a good – where we share information both directions and there’s a level of trust built up between the professor and us, and between us and the university […] And also we’re working with our global [headquarters] partners […] So there has to be a lot of trust and very strong communication to make things work. So part of our role is liaison, as well.” {251, industry, Asia-Pacific}  “…and then just availability and open communication, so being able to text anytime and know that you'll get a response and can come up with a plan together, I'd say those are the three biggest enablers, so, values, shared vision and open communication.” {267, nonprofit, Asia-Pacific}  “All the organizations come together for a morning huddle before their day starts […] ‘Where’s that patient at, who’s doing what today, any emerging issues, have we heard what’s working what’s not working?’ And we actually develop from an individual consciousness to a shared collective consciousness – and we also developed the first integrated EMR [in this region], across [several] organizations.” {237, nonprofit, North America}  “[Our partners] need to be comfortable accepting [our discharged] patients with [such difficult] conditions – because often people are very scared. So when some of our patients [might suddenly take a turn for the worse] and they are not equipped – and they don’t have [specialists] to manage the patients and this becomes a scary thing for the [partners] so what we did is we offer very strong support. We regularly send our doctors to do ward rounds, to case conference, and we have virtual consultations and we basically keep the communication channel open.” {238, government, Asia‑Pacific} |
| Theme  Understanding where other partners are coming from  e.g.,  - consideration of other partners’ perspectives, frames of reference  - with benefit of respect, insight, listening, familiarity  - as a basis to engage effectively | Illustrative quotes  “We’re all very comfortable with taking the time to understand where each of us is coming from and why we might have come to a particular conclusion in terms of our own position on a particular issue.” {234, nonprofit, EU+}  “..and had they thought about that? You know, appeal to the good conscious and professionalism [of the physicians] and basically hit home with a lot of evidence and think about the psychology of the parties […] The main drives of physician behavior are actually inherent competition, professionalism, a sense of professionalism, a sense of logic – that’s their whole upbringing, interest in problem solving. […] It was a tactic to not only help us be able to have reasonable and rational passion-light evidence-heavy debates whereas in the past my observation was passion-heavy evidence-light debates…”  {236, government, North America}  “And I think thirdly we had spent by that point two years together as a joint team – and I think once we started to listen, ‘What are the real drivers, […] this is what the biotech wants, these are their drivers.’ […] The how of getting through that period was really about listening…” {220, industry, EU+}  “[In addition to my role as a healthcare executive] I’m also an [elected local politician] and I’m also a [community] leader. […] So I’m talking about healthcare, people who need help [to politicians and community leaders] talking about social bonding. So when you have that different perspective, even though the beneficiaries might be the same, you have that bit of clash and so it’s like, ‘Hmm, this person is taking advantage of me, why should I help them?’ I think the advantage of wearing different hats [as I do] is when you think to yourself and you say*, ‘Right!’* (as in, *‘ah ha!’*) So that’s it, you see […] it’s no more about me wanting my job done by you – it’s about you having [a community] activity and how can I come in and add on to it. So I think we all want to do good but what is the language we use and how can we fit into each others’ activities rather than saying, ‘This is what I want you to help me on.’ Rather I say, ‘Hey, yes, that’s a great activity – I can add more to it, you know, I can provide additional activities for you…”  {205, government, Asia-Pacific} |
| Theme  Useful convenings of partners, regular check-ins  e.g.,  - focused in-person convenings to help advance complex joint work (working sessions, summits, etc)  - periodic meetings, calls | Illustrative quotes  “..the success factor in that partnership was where we actually had a good and long and very many days together with a specific team that they flew in and we had the team from the hospital and they had the time to discuss what gaps do they have in their knowledge. […] You could match a [specialist hospital] physician with a [company] technician in intervention procedures. They could really look into things together in different angles.”  {259, government, EU+}  “..absolutely making sure that you brought people together face to face, to work through the complexities of the work that they were doing…” {260, nonprofit, Asia‑Pacific}  “..the [quarterly] check-ins is really important, to deliberately make space to talk about the partnership is really important for the relationship management…” {216, nonprofit, EU+} |
| **Organizing theme**  **Active management, from early on** |  |
| Theme  Assiduous management efforts to set the stage for success, to follow‑through and deliver    e.g.,  - pragmatic, diligent attention to what needs to be done  - in beginning, to lay groundwork, foundations  - over time, on joint plans, on commitments to other partners | Illustrative quotes  “Just putting in the time early, and setting the right stage and setting the right tone, have proven invaluable to us. […] The general thing is early in the partnership you just want to create everything, to solve every problem in the world – but if you don’t take into account the more boring stuff it’s not going to be a fruitful partnership.” {259, government, EU+}  “..and [the partners] wouldn’t do any kind of programming or resource allocation without the governance, vision, communications, measurement – they just did everything you do to build a sturdy foundation if you’re going to play at that level.” {229, nonprofit, North America}  “In this case there was a very pragmatic approach from the beginning, about tangible deliverables, things we knew could be accomplished – and then bringing some of the emphasis that industry has on project management, achieving timelines, staying on budget, that was very important. […] You need the vision on one side, but you need to have very pragmatic steps […] that you drive to.” {246, industry, EU+}  “Over the years we built very strong partnership with these people. […] We discharge our patients to these facilities. However sometimes you might never know that a patient who’s recovering can become acute again […] and we will go in and tell them, ‘We are here for you because you supported us (i.e., by accepting our patients and the special challenges they entail) and it’s our job to support you.’ In the past there were some unfortunate [patient safety] events – for example […] so we actually went in and went through a whole root-cause analysis and we told them, ‘Going forward, this is what we are going to do’ – so they were very appreciative of our help. So we have very strong connections and partnership with [these facilities].”  {238, government, Asia-Pacific}  “..he’s able to pull out what he can do as part of the system, what they can commit to […] and most importantly [get that] to his staff because if he sets expectations they need to deliver…” {267, nonprofit, Asia-Pacific} |
| Theme  Clearly define how partners will work together, joint objectives  e.g.,  - through agreements, rules of engagement, guiding principles  - in terms of overall aims, vision, governance, specific outcomes, deliverables, responsibilities  - discussed and agreed by leaders, via facilitated processes | Illustrative quotes  “..whenever we partner with someone we actually have a formal agreement called a [partnership] agreement. There’s no money being – sometimes there’s money but it’s not the reason we have a contract – but it’s really to have absolute clarity about what the mutual value exchange is, the deliverables we’re all agreeing to, and how we’re going to work together. The biggest thing is that sets things up for success – sorting all that stuff very deliberately upfront...” {216, nonprofit, EU+}  “We had a big summit where the most senior leaders of the organizations got together and talked about what we want to accomplish together and what we’re committed to. […] We wanted to figure out exactly what working together means, because it’s all well and good to talk about it in theory but to figure out exactly what it means, what we’re going to do, […] find common ground, and agree on leading indicators and mid-term outcomes, that kind of stuff [made] us both feel comfortable.” {253, industry, North America}  “..we [moved] into a results agreement with each partner, specific business plan with each partner and keeping a completely different mechanism for accountability. And initially the partners felt this was a kind of micromanagement from a body that was supposed to serve rather than manage the partners. But then it was well understood it was actually for the best intention of achieving the outcomes and [those changes] worked quite well.” {219, industry, EU+}  “So we brought together [the partner] organizations including the providers within those organizations, so traditional leadership and then frontline leadership, to […] co‑define a shared purpose, a set of [guiding] principles by which we advance and […] I would say that is the most important thing in anything I do – it's the approach to successful partnerships.”  {237, nonprofit, North America} |

**Note on rights and permissions** The original authors of this document (“Additional file 3: Success factors and illustrative quotes”) are Greg Zwisler, Christopher Sauer, and David Shoultz. The original source, for citation purposes, is their manuscript entitled “Vital lessons from struggling partnerships and potential partnerships: an international study with leaders across the health sector”, published by BMC Health Services Research. This Additional file and its contents are licensed under a Creative Commons Attribution 4.0 International License, which permits use, sharing, adaptation, distribution and reproduction in any medium or format, as long as you give appropriate credit to the original author(s) and the source, provide a link to the Creative Commons licence, and indicate if changes were made. To view a copy of this licence, visit <http://creativecommons.org/licenses/by/4.0/>.
