# Supplementary material for: Vital lessons from struggling partnerships and potential partnerships: an international study with leaders across the health sector
Source: BMC Health Serv Res. 2024 Nov 26;24:1470. doi: 10.1186/s12913-024-11944-7 (PMC11590265; doi:10.1186/s12913-024-11944-7)
Supplement: Supplementary file 4 — Supplementary Material 4. [file 12913_2024_11944_MOESM4_ESM.docx]

**Additional file 4: More on negative factors’ contributions to literature**

About one third of negative factors identified as themes were previously not recognized in the literature this study addressed, generally or with respect to some of their main aspects (see manuscript Background for delineation of the literature this study addressed). The remainder were recognized variously among reviews (1–5), for example, which also noted other negative factors and/or ‘barriers’, a similar term used in the literature. Negative factors previously not recognized in the literature clustered under three organizing themes and are discussed below, with notes distinguishing previously unrecognized aspects of themes as necessary.

**Alignment time-consuming to establish and uncertain**: Most themes under this organizing theme were previously not recognized, namely:

- ‘Negotiations, alignment building, contracting involved seen as: difficult, prolonged, vulnerable to failure, too time and resource intensive’.
- ‘Turnover of senior leaders, partnership leads, liaisons at partners’: in particular, turnover of senior leaders such as CEOs, with consequent disruptions to alignment. Turnover of partnership managers and effects on coordination have been recognized (2,3,6).
- ‘Too many partners involved’: reflected concerns with varying numbers of partners from 3 to ≥6; except an unpublished report cited in (7), previous reports’ most comparable cautions concerned a diversity of partners and specifically partners’ heterogeneity, not quantity (5,8,9).
- ‘Resistance, limited cooperation from within partner’s organization and structure’: in particular, regarding internal structures and conflicting interests, and related limitations of interlocutors’ positions and authority. Previous reports noted frontline reactions to implementation (1,3,10) and unsupportive leadership (3,11,12).
- ‘Cumbersome pace, processes of universities, major corporations, government bodies’: in particular, with respect to major corporations.

**Challenges of efforts involving newer or less familiar areas and approaches**: Most themes under this organizing theme were previously not recognized, specifically:

- ‘Risk-averse attitudes, people afraid to try something new’: while reviews have noted resistance to change (1,12), this sub-theme was broadly concerned with overcaution and hesitation to try new things, use trial and error, and accept some risk.
- ‘Business models uncertain, yet to be determined, inadequate’.
- ‘Interested and suitable partners hard to identify, not aware of each other’: previously not recognized beyond the limited case of a clinician who said they did not necessarily understand how other organizations in their community contributed to care (1,13).
- ‘To yield results only in longer term with notable uncertainties, upfront investments involved’.

**Problematic health-sector structures and mentalities**: Two themes under this organizing theme were previously not recognized:

- ‘Paternalism, traditional mindsets limit meaningful patient involvement’.
- ‘Insularity, not going outside health sector or home country to learn from, work with others’.

While reviews have mentioned organizational and professional cultures, for instance, they have not done so concerning paternalism or insularity (2,3).

Finally, two other themes were previously not recognized:

- ‘Alignment not given more probing, careful attention upfront’.
- ‘Treated like a vendor rather than a partner’.

**Note on rights and permissions** The original authors of this document (“Additional file 4: More on negative factors’ contributions to literature”) are Greg Zwisler, Christopher Sauer, and David Shoultz. The original source, for citation purposes, is their manuscript entitled “Vital lessons from struggling partnerships and potential partnerships: an international study with leaders across the health sector”, published by BMC Health Services Research. This Additional file and its contents are licensed under a Creative Commons Attribution 4.0 International License, which permits use, sharing, adaptation, distribution and reproduction in any medium or format, as long as you give appropriate credit to the original author(s) and the source, provide a link to the Creative Commons licence, and indicate if changes were made. To view a copy of this licence, visit <http://creativecommons.org/licenses/by/4.0/>.

References

1. Auschra C. Barriers to the integration of care in inter-organisational settings: A literature review. Vol. 18, International Journal of Integrated Care. 2018.

2. Alderwick H, Hutchings A, Briggs A, Mays N. The impacts of collaboration between local health care and non-health care organizations and factors shaping how they work: a systematic review of reviews. BMC Public Health. 2021;21(1).

3. Lowther HJ, Harrison J, Hill JE, Gaskins NJ, Lazo KC, Clegg AJ, et al. The effectiveness of quality improvement collaboratives in improving stroke care and the facilitators and barriers to their implementation: a systematic review. Vol. 16, Implementation Science. 2021.

4. Rybnicek R, Königsgruber R. What makes industry–university collaboration succeed? A systematic review of the literature. J Bus Econ. 2019;89(2).

5. Seaton CL, Holm N, Bottorff JL, Jones-Bricker M, Errey S, Caperchione CM, et al. Factors That Impact the Success of Interorganizational Health Promotion Collaborations: A Scoping Review. Am J Heal Promot. 2018;32(4).

6. Bryson JM, Crosby BC, Stone MM. Designing and Implementing Cross-Sector Collaborations: Needed and Challenging. Public Adm Rev. 2015;75(5).

7. Woulfe J, Oliver TR, Zahner SJ, Siemering KQ. Multisector partnerships in population health improvement. Prev Chronic Dis. 2010;7(6).

8. Lasker RD, Weiss ES, Miller R. Partnership Synergy: A Practical Framework for Studying and Strengthening the Collaborative Advantage. Milbank Q. 2001;79(2).

9. Poulos RG, Donaldson A, McLeod B. Developing injury prevention policy through a multi-agency partnership approach: A case study of a state-wide sports safety policy in New South Wales, Australia. Int J Inj Contr Saf Promot. 2012;19(2).

10. Aunger JA, Millar R, Greenhalgh J, Mannion R, Rafferty AM, McLeod H. Why do some inter-organisational collaborations in healthcare work when others do not? A realist review. Syst Rev. 2021;10(1).

11. Ankrah S, AL-Tabbaa O. Universities-industry collaboration: A systematic review. Scand J Manag. 2015;31(3).

12. Zamboni K, Baker U, Tyagi M, Schellenberg J, Hill Z, Hanson C. How and under what circumstances do quality improvement collaboratives lead to better outcomes? A systematic review. Vol. 15, Implementation Science. 2020.

13. Tsasis P, Evans JM, Owen S. Reframing the challenges to integrated care: A complex-adaptive systems perspective. Int J Integr Care. 2012;12(JULY-SEPTEMBER 20).

14. Petchel S, Gelmon S, Goldberg B. The organizational risks of cross-sector partnerships: A comparison of health and human services perspectives. Health Aff. 2020;39(4).

15. Gazley B. The Current State of Interorganizational Collaboration: Lessons for Human Service Research and Management. Vol. 41, Human Service Organizations Management, Leadership and Governance. 2017.

16. Barnett J, Vasileiou K, Djemil F, Brooks L, Young T. Understanding innovators’ experiences of barriers and facilitators in implementation and diffusion of healthcare service innovations: A qualitative study. BMC Health Serv Res. 2011;11.

17. Mattessich PW, Johnson KM. Collaboration: What Makes It Work, 3rd Edition. Nashville: Fieldstone Alliance; 2018.

18. Ford KL, Portz JD, Zhou S, Gornail S, Moore SL, Zhang X, et al. Benefits, Facilitators, and Recommendations for Digital Health Academic-Industry Collaboration: A Mini Review. Front Digit Heal. 2021;3.

19. Butterfoss F. Coalitions and partnerships in community health. San Francisco: Jossey-Bass; 2007.

20. Weishaar H, Collin J, Amos A. Tobacco control and health advocacy in the European Union: Understanding effective coalition-building. Nicotine Tob Res. 2016;18(2).
